# Phosphorylated exogenous alpha-synuclein fibrils exacerbate pathology and induce neuronal dysfunction in mice

**DOI:** 10.1038/s41598-017-15813-8

**Published:** 2017-11-28

**Authors:** Mantia Karampetsou, Mustafa T. Ardah, Maria Semitekolou, Alexia Polissidis, Martina Samiotaki, Maria Kalomoiri, Nour Majbour, Georgina Xanthou, Omar M. A. El-Agnaf, Kostas Vekrellis

**Affiliations:** 1 Center of Basic Research, Biomedical Research Foundation of the Academy of Athens, Athens, 11527 Greece; 2 Division of Human and Animal Physiology, Department of Biology, National and Kapodistrian University of Athens, Panepistimiopolis, 15784 Athens, Greece; 3 Department of Biochemistry, Faculty of Medicine and Health Sciences United Arab Emirates University Al Ain- UAE, Al Ain, 15551 UAE; 4 Cellular Immunology Laboratory, Division of Cell Biology, Centre for Basic Research, Biomedical Research Foundation of the Academy of Athens, Athens, 11527 Greece; 5 Center of Clinical, Experimental Surgery & Translational Research, Biomedical Research Foundation of the Academy of Athens, Athens, 11527 Greece; 6 Biomedical Sciences Research Center ‘Alexander Fleming’, Fleming 34, Vari, 16672 Greece; 7 Qatar Biomedical Research Institute (QBRI), and College of Science and Engineering, Hamad Bin Khalifa University (HBKU), Qatar Foundation, Doha, Qatar

## Abstract

Approximately 90% of alpha-synuclein (α-Synuclein) deposited in Lewy bodies is phosphorylated at serine 129 suggesting that the accumulation of phosphorylated α-Synuclein is critical in the pathogenesis of Parkinson’s disease. However, *in vivo* experiments addressing the role of phosphorylated α-Synuclein in the progression of Parkinson’s disease have produced equivocal data. To clarify a role of Ser129 phosphorylation of α-Synuclein in pathology progression we performed stereotaxic injections targeting the mouse striatum with three fibrilar α-Synuclein types: wt-fibrils, phosphorylated S129 fibrils and, phosphorylation incompetent, S129A fibrils. Brain inoculation of all three fibrilar types caused seeding of the endogenous α-Synuclein. However, phosphorylated fibrils triggered the formation of more α-Synuclein inclusions in the Substantia Nigra pars compacta (SNpc), exacerbated pathology in the cortex and caused dopaminergic neuronal loss and fine motor impairment as early as 60 days post injection. Phosphorylated fibril injections also induced early changes in the innate immune response including alterations in macrophage recruitment and IL-10 release. Our study further shows that S129 phosphorylation facilitated α-Synuclein fibril uptake by neurons. Our results highlight the role of phosphorylated fibrilar α-Synuclein in pathology progression *in vivo* and suggest that targeting phosphorylated α-Synuclein assemblies might be important for delaying inclusion formation.

## Introduction

α-Synuclein is the main protein component of Lewy bodies (LBs), the major pathological hallmarks of Parkinson’s disease and other synucleinopathies. α-Synuclein is biochemically and genetically linked to Parkinson’s disease^1,2^. Nearly all α-Synuclein accumulated within LBs is phosphorylated on serine 129 (Ser-129)^3–5^ but the significance of phosphorylation in the biology and pathophysiology of the protein is still controversial. Although the phosphorylation state of α-Synuclein appears to influence its aggregation propensity and toxicity^6^, it is still not known whether phosphorylation promotes or prevents the aggregation and toxicity of α-Synuclein*. In vitro* and *in vivo* studies, examining phosphorylation of α-Synuclein on different sites have resulted in equivocal results, showing promotion^7^ or inhibition or no effect on inclusion formation^8,9^. The use of α-Synuclein mutants to either prevent or mimic phosphorylation in various *in vivo* models has also produced conflicting results^10–12^. A number of *in vivo* studies have demonstrated the ability of α-Synuclein fibrilar species to be secreted and uptaken from neuronal cells in a trans-synaptic function. These fibrilar forms (PFF) can initiate the conversion of normal endogenous protein to pathogenic aggregated form, hyper-phosphorylated at Ser129 in the brain of rodents. A theory is emerging that α-Synuclein fibrils behave as prion-like strains, each with distinct structural and biochemical features^13^. Transmission of pathology is verified by the presence of phosphorylated α-Synuclein in interconnected brain regions. However, the effect, if any, of the phosphorylation of α-Synuclein fibrils in the seeding and pathological accumulation of α-Synuclein has not been examined. Mice injected with wt α-Synuclein pre-formed fibrils (PFF) exhibited cell death 90 days post injection (dpi)^14^. Here we chose to examine the effect of phosphorylated α-Synuclein pre-formed fibrils (P-PFF) at the early time point of 60 dpi so as to elucidate early events in disease pathology. We show, that in wt mice intracerebral injections of P-PFF induced a robust formation of pathological inclusions and triggered dopaminergic neuronal loss and motor symptoms in inoculated animals. Interestingly, exacerbated pathology upon *in vivo* administration of P-PFF was associated with an altered innate immune response early post injection. This was reflected by a decreased recruitment of CD45^+^CD11b^+^ macrophages from the peripheral lymphoid compartment, along with a significant decreased release of the anti-inflammatory cytokine IL-10 in the striatum. Importantly, we found that P-PFF are more efficiently uptaken by neurons and lead to increased seeding and accumulation of the endogenous α-Synuclein.

## Results

### Characterization of human α-Synuclein fibrils

**Figure 1:**
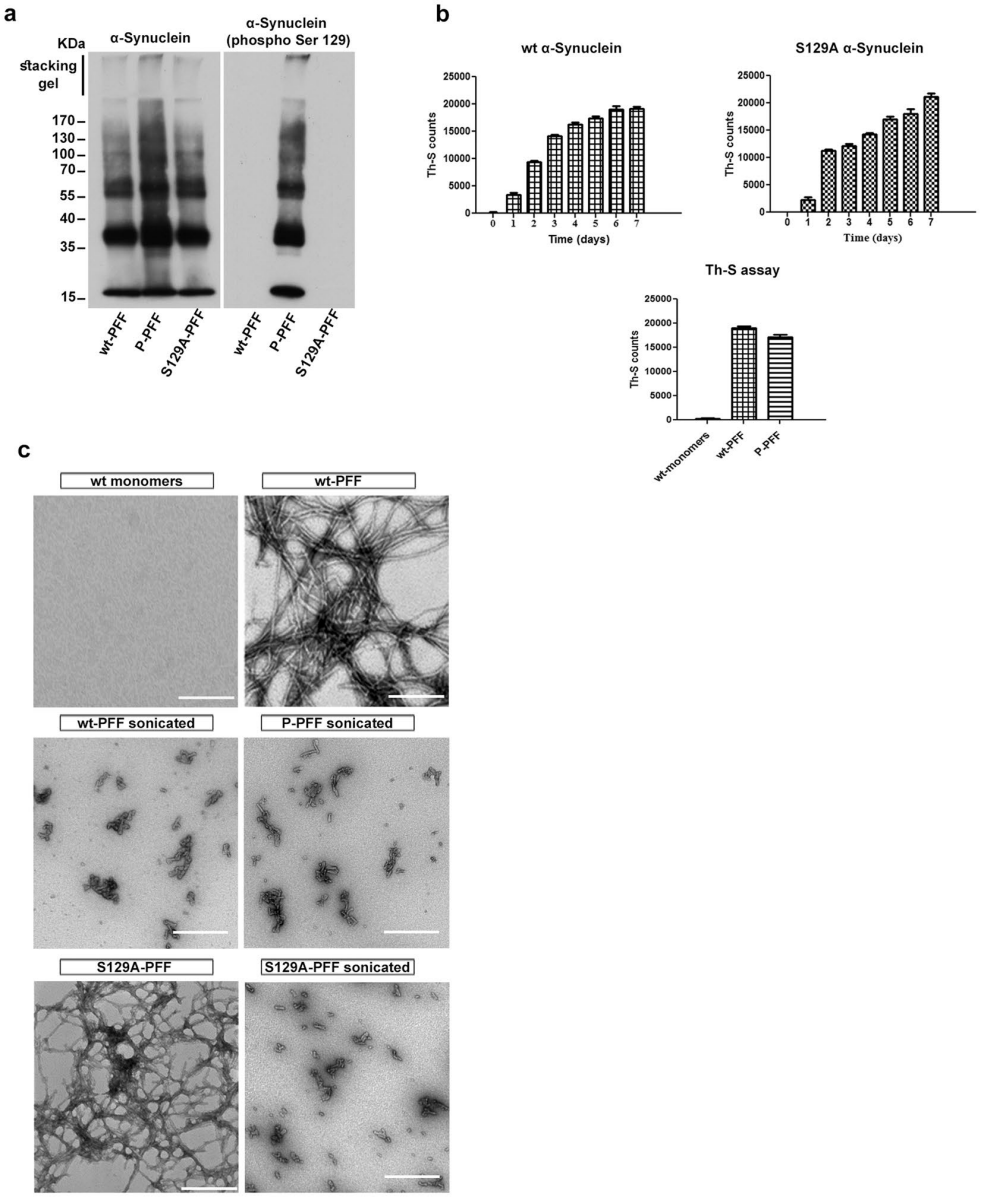
Characterization of wt-, P- and S129A- PFF. (**a**) Western blotting analysis for wt-, P- and S129A-PFF. Equal amounts of fibrils were analyzed in a 10% SDS-PAGE gel using the C20 antibody. P-PFF were detected with the α-Synuclein (phospho Ser) antibody whereas no signal was observed for the S129A- and wt-PFF. (**b**) Fibril formation monitoring by Th-S assay. Graphs show: fibril formation monitoring of the wt- and S129A-monomers incubated for 7 days (top). Comparison of the fibril content of wt-PFF and P-PFF (bottom). Monomeric α-Synuclein was used as control. The assays were performed in triplicate. (**c**) Electron microscopy images of negatively stained samples of the different types of α-Synuclein to confirm the presence of fibrils compared to the monomeric non-fibrilar α-Synuclein. *Scale bar*, 500 nm.

We initially generated crude wt-PFF, which were subsequently sonicated and phosphorylated. P-PFF were thus prepared by phosphorylation of the sonicated wt-PFF. We chose this approach so as to ensure minimal structural differences between the phosphorylated and wt fibrils. Immunoblotting was used to confirm the presence of high molecular weight species after incubation of the recombinant α-Synuclein monomers for 7 days at 37 °C. As shown in Fig. 1a, α-Synuclein species with different molecular weights were readily formed by all types of PFF. The effective phosphorylation of P-PFF was confirmed by immunoblot analysis using an antibody that recognizes hyper-phosphorylated α-Synuclein (phospho Ser129). High and low molecular weight phosphorylated species were detected after *in vitro* phosphorylation of wt-PFF. As expected, S129A-PFF, which lack the S129 residue and wt-PFF were not detectable with the α-Synuclein (phospho Ser129) antibody (Fig. 1a). The specific phosphorylation of P-PFF at S129 position was further verified by Mass Spectrometry analysis (MS). Following AspN digestion, two differentially cleaved mono-phosphorylated α-Synuclein peptides were identified in its C-terminus. The sequence of the peptides and the variable modifications of each are shown in the Supplementary Table. Both peptides contained the serine at position 129 of the protein as well as tyrosine residues. The analysis of the corresponding MS/MS spectra (Supplementary Fig.S1) could confidently localize the phospho group to the S129 (>99%). In addition, the non-phosphorylated counterpart was not detectable indicating that the majority of the peptide is phosphorylated (Supplementary Table). To further characterize the phosphorylation state of our P-PFF we used a novel “in house” monoclonal antibody (A4B12) that specifically recognizes the non-phosphorylated form of α-Synuclein and does not cross react with S129 phosphorylated form (Omar El-Agnaf, in preparation). As shown in Supplementary Fig.S2, only a small fraction of the P-PFF generated is non-phosphorylated at S129. Th-S assay was used to monitor the fibril formation of different types of α-Synuclein (Fig. 1b). Samples of recombinant wt and S129A α-Synuclein that had been incubated for 7 days, showed a similar trend in forming fibrils. P-PFF and wt-PFF fibrils also appear similar by Th-S counts (Fig. 1b). The recombinant wt α-Synuclein monomers were used as negative control. Electron microscopy was also used to monitor the fibril morphology and to confirm the presence of fibrils (Fig. 1c).

### Exogenous α-Synuclein fibrils induce pathologic inclusions early post-injection

**Figure 2:**
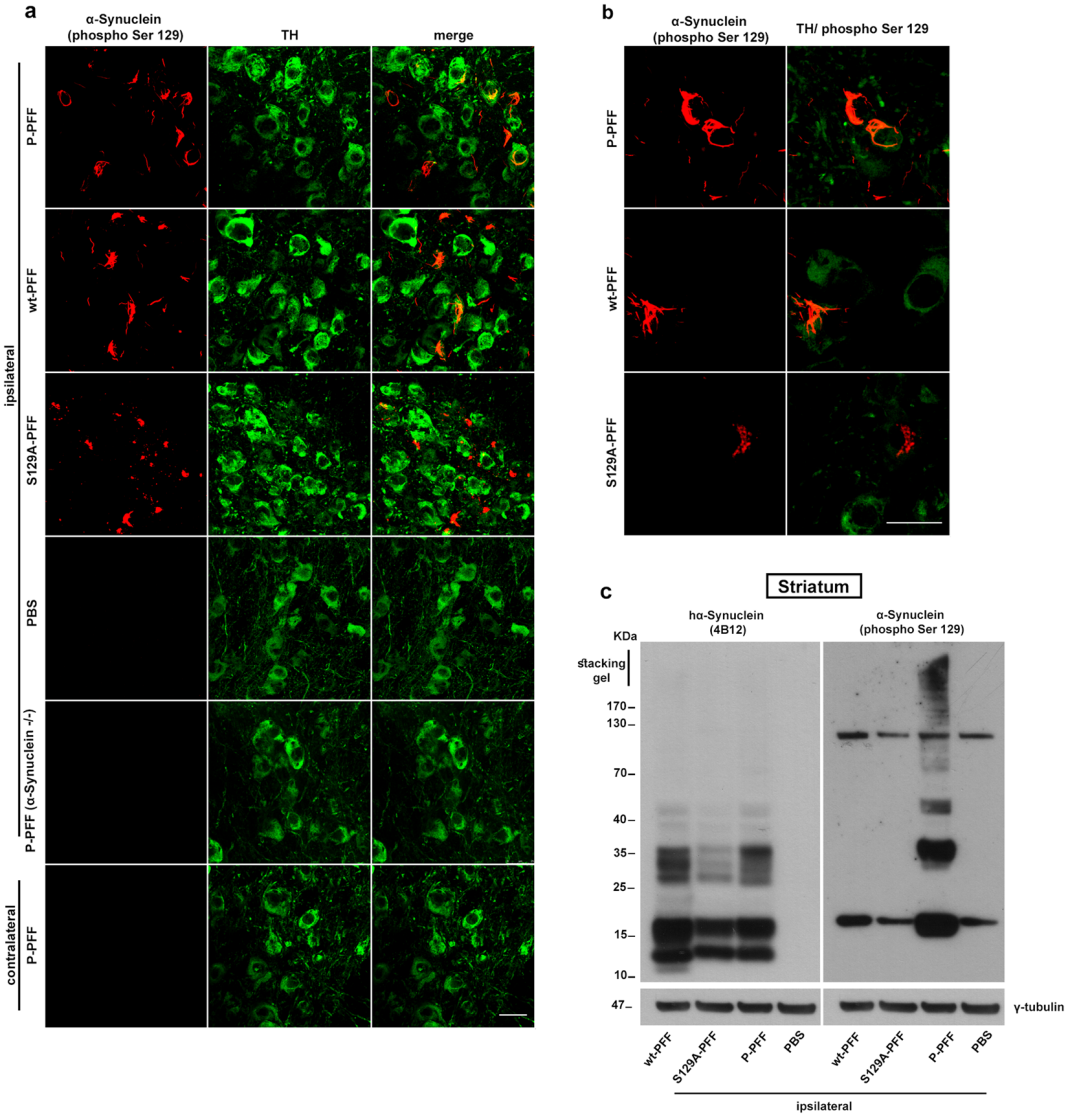
Pathological α-Synuclein accumulation in the SNpc dopaminergic neurons of wt mice following stereotaxic unilateral striatal injection of three different human-PFF types (P-PFF, wt-PFF, and S129A-PFF). Animals were analyzed 60 dpi. (**a**) Confocal images showing double immunostaining for P-α-Synuclein and TH in nigral sections of PFF-injected animals. Accumulation of hyper-phosphorylated α-Synuclein (α-Synuclein phospho Ser 129) is evident in dopaminergic neurons (TH) of the ipsilateral SNpc. Pathology is absent in the ipsilateral side of PBS injected animals. The contralateral side of P-PFF injected animals shows no signs of pathologic accumulations. α-Synuclein (phospho Ser129) immunoreactivity is not detected in the ipsilateral nigra of α-Synuclein null (−/−) animals injected with P-PFF (n = 4). (**b**) Images in higher magnification are showing P-α-Synuclein accumulations induced by the different types of PFF. (**c**) Striatal tissue of injected animals (3 dpi) was extracted and immunoblotted with the 4B12 and phospho Ser 129 antibodies. Human α-Synuclein was readily detected in the striatal extracts. S129A- and wt-PFF could not be detected with the phospho Ser 129 antibody. γ-tubulin was used as a loading control (cropped gel/blot is shown). Scale bars represent 25 μm.

To gain insight into the role of phosphorylation and its ability to template pathology of α-Synuclein *in vivo* we performed stereotaxic injections targeting the mouse right dorsal striatum. Equal numbers of male and female wt and α-Synuclein null mice (α-Synuclein −/−), 2–4 month-old, were inoculated with 4,25 μg of human recombinant fibrilar α-Synuclein of three different types: a) wt-PFF, b) P-PFF which are stably phosphorylated at residue serine 129 and c) mutant S129A-PFF that bear the substitution serine (S) to alanine (A) and are thus incapable of de novo phosphorylation at this site. To evaluate the potential of different fibrilar types of α-Synuclein to seed pathology we chose the early time point of 60 days post injection (dpi) for our analyses. 60 dpi midbrain sections were analyzed by immunohistochemistry using an antibody that recognizes hyper-phosphorylated α-Synuclein (phospho Ser129). With all three different fibrilar types injected we were able to detect cytoplasmic accumulations of P-α-Synuclein surrounding the nucleus of tyrosine-hydroxylase (TH) positive neurons in the SNpc (Fig. 2a). 3D reconstruction analysis of the sections confirmed the cytoplasmic distribution of the inclusions in TH-stained neurons (Supplementary video file). These pathological accumulations were evident in all PFF-injected animals (Fig. 2a,b). No pathological accumulations were observed in PBS-injected animals or in the contralateral SNpc (Fig. 2a). In agreement with previous reports^15^, we could not detect any P-α-Synuclein positive staining in PFF-injected α-Synuclein null animals (Fig. 2a). To ascertain that the recombinant PFF were unable to be phosphorylated *in vivo*, animals were injected with the three different types of fibrils and the striatum was excised 3 dpi. Western blotting with the human α-Synuclein specific (4B12) and the α-Synuclein (phospho Ser129) specific antibodies revealed that neither the wt- nor the mutant S129A fibrils could be phosphorylated *in vivo* at this early time point (Fig. 2c).

### PFF induce endogenous α-Synuclein accumulations which are fibrilar in nature

**Figure 3:**
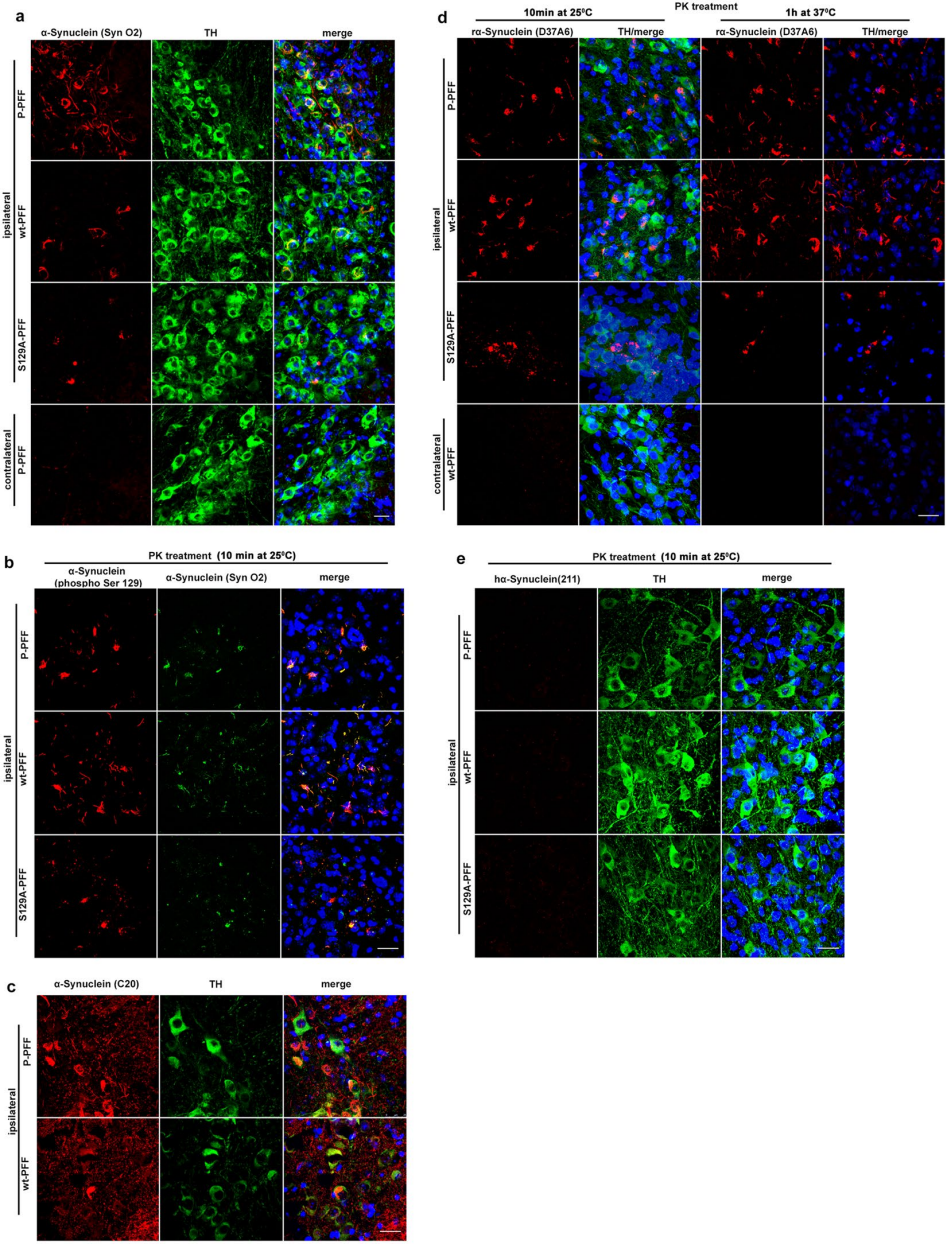
Characterization of SNpc intraneuronal α-Synuclein accumulations. (**a**) Double labeling with the conformational specific α-Synuclein antibody SynO2 and TH is showing the fibrilar nature of the α-Synuclein cytoplasmic accumulations restricted to TH neurons of the ipsilateral SNpc following injections with P-, wt- and mutant S129A-PFF at 60 dpi. Contralateral side shows only background staining with the SynO2 antibody. (**b**) Representative sections of SNpc from all PFF injected animals showing the co-staining of α-Synuclein accumulations with the α-Synuclein (phospho Ser 129) and SynO2 antibodies following PK treatment for 10 min at 25 °C to expose antigenic sites. (**c**) α-Synuclein accumulations also stained positive with the C20 antibody. (**d**) Host α-Synuclein expression is essential for the formation of pathological α-Synuclein accumulations. TH-stained nigral sections (PK-treated, 10 min at 25 °C) are exhibiting α-Synuclein accumulations that are positive for the endogenous rodent α-Synuclein (D37A6) antibody. PK resistant D37A6-positive accumulations were also evident following prolonged PK treatment (1 h at 37 °C) in nigral sections until the TH signal is not detectable (**e**) α-Synuclein accumulations do not stain with the human specific anti-α-Synuclein (211) antibody. TO-PRO-3 (blue) was used as a cell nuclear marker (n = 4). Scale bars represent 25 μm.

To further characterize the observed α-Synuclein accumulations, we performed immunohistochemistry with various α-Synuclein specific antibodies. Nigral sections were stained for α-Synuclein using the conformational specific antibody SynO2 that recognizes aggregated structures of full length α-Synuclein (fibrilar and oligomeric)^16^ (Fig. 3a). Following Proteinase K (PK) treatment (10 min at 25 °C), pathological α-Synuclein accumulations were stained positive with antibodies to α-Synuclein (phospho Ser 129) and SynO2 (Fig. 3b), suggesting that all three types of injected fibrils are potent to induce α-Synuclein pathology. Moreover, α-Synuclein lesions were also detected by immunostaining with the C20 antibody, which recognizes both human and endogenous mouse α-Synuclein (Fig. 3c). To further investigate the nature of these abnormal α-Synuclein accumulations the sections were stained with species-specific antibodies. PK-treated sections (10 min at 25 °C) were stained with the rodent specific antibody D37A6 (Fig. 3d). To evaluate further the extent of α-Synuclein aggregation in the injected mouse brains, we performed immunohistochemical analyses on sections treated with PK (1 h at 37 °C), which allows the detection of highly ordered PK resistant accumulations. PK-treated sections stained positive for the D37A6 antibody, confirming the fibrilar nature of the inclusions (Fig. 3d and Supplementary Fig. S3). No signal was detected using two human specific antibodies, 211 (Fig. 3e) and 5C2 (not shown). Our experiments demonstrate that the endogenous rodent α-Synuclein is a major component of the abnormal pathological accumulations. As expected, these α-Synuclein inclusions also co-localized with ubiquitin and p62, known markers for LB-like inclusions (Supplementary Fig. S4).

### Phosphorylated PFF promoted pathological α-Synuclein accumulations in the SNpc and impaired motor coordination in injected animals

**Figure 4:**
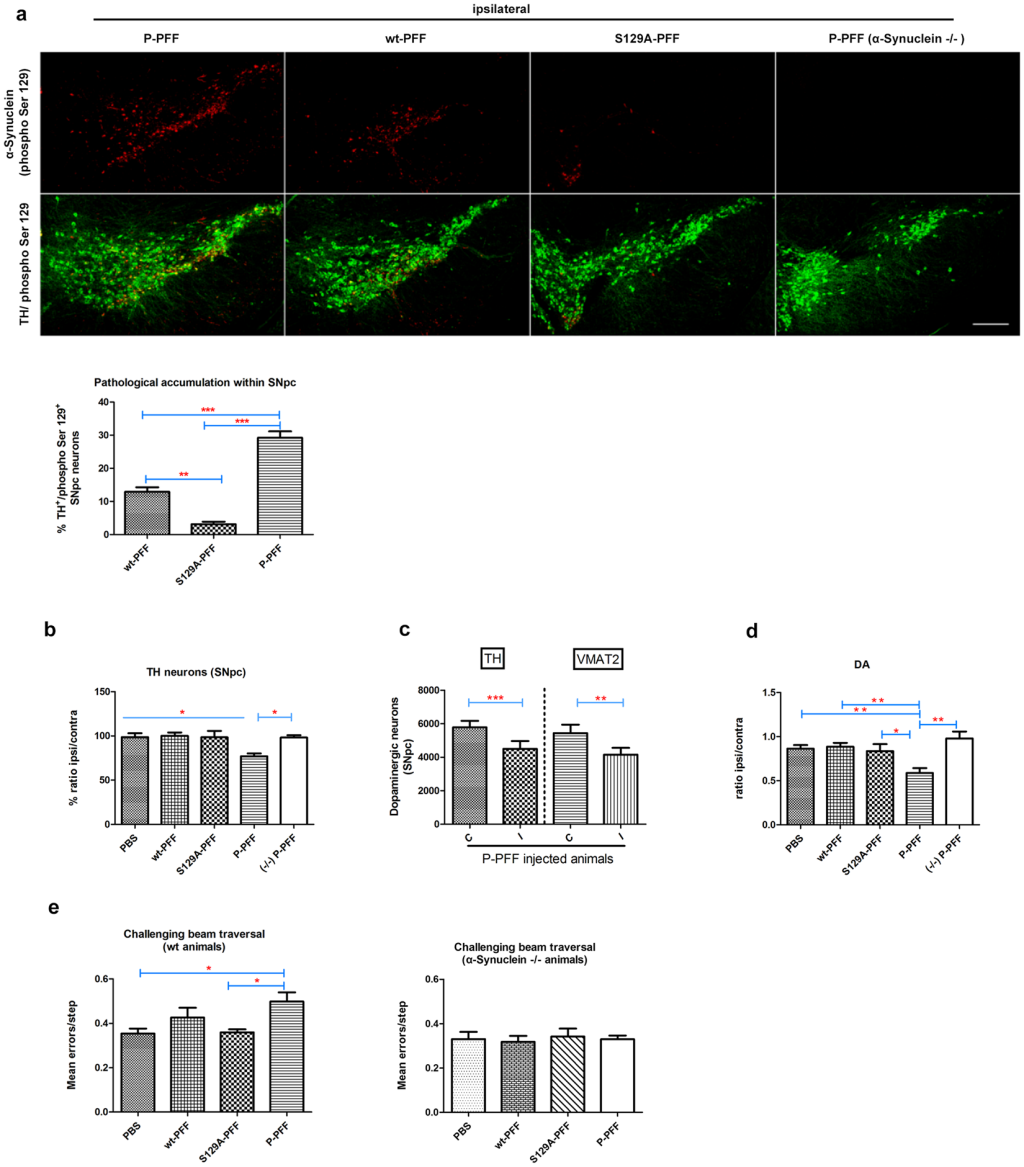
P-PFF exacerbate the pathology within the SNpc and significantly impair the integrity of the dopaminergic neurons. (**a**) Coronal nigral sections were immunostained for α-Synuclein (phospho Ser S129) and TH. The absolute numbers of P-accumulations that were formed within the TH positive neurons in the different fibril-injected brains were counted. As shown in representative tiled images for each of the P-, wt-, and S129A-PFF injected animals, P-PFF induced a more widespread pathology compared to the other two fibrilar types. P-PFF injected α-Synuclein null mice did not show any sign of pathology. Graph depicts the percentage of TH neurons containing P-α-Synuclein positive accumulations for each treatment group (n = 4 animals per group, 3 sections per animal). (**b**) Stereological analysis of TH-positive neurons is showing a significant loss of dopaminergic neurons in P-PFF injected animals compared to the PBS, wt-, S129A-PFF injected animals and to P-PFF α-Synuclein null (−/−) injected animals. The data are presented as a percentage of ipsilateral to contralateral side (n = 5–6 animals per group). (**c**) Decreased nigral TH positive neuron number was confirmed with VMAT2 stereological analysis following P-PFF injections (4–6 animals per group, paired Student’s t-test analysis). (**d**) Significant decrease in striatal DA levels in wt animals injected with P-PFF. The data are presented as a ratio of ipsilateral to contralateral side (n = 5–7 animals per group). (**e**) Fine motor impairment as increased errors/step in the challenging beam traversal test in P-PFF- vs. S129A- and control PBS- injected animals (n = 7–8 animals/group). Similar injections did not cause any motor impairment in null α-Synuclein (−/−) mice (n = 5 animals/group). Data represent mean values ± SEM. Differences were estimated using one-way ANOVA followed by Tukey’s post-hoc test. (**a**) p < 0,0001 (**b**) p = 0,0052 (**c**) For TH p = 0,0002, and VMAT p = 0,0062 two-tailed paired t-test (**d**) p = 0,0009 (**e**) p = 0,0163. Scale bar in (**a**) represents 250 μm.

For mapping pathological accumulations within the SNpc, coronal sections from each animal (see Materials and Methods), were immunostained for α-Synuclein (phospho Ser129). Separated images were tiled using the confocal microscope’s automated stage and processed for counting the absolute numbers of P-accumulations that were formed within the TH positive neurons in the different PFF-injected brains. Interestingly, the number of the pathological inclusions appeared to differ significantly depending on the type of fibril injected. P-PFF injected animals accumulated more inclusions within the TH positive neurons at the given time point compared to the wt- or S129A-PFF injected animals (29,2 ± 1,9% vs 12,9 ± 1,3% vs 3,1 ± 0,7% respectively). P-PFF-injected α-Synuclein null mice did not show any sign of pathology (Fig. 4a). Wt- and S129A-injected α-Synuclein null mice also did not exhibit any pathology (not shown). To assess the effect of the different fibrilar types on the integrity of the SNpc, stereological analysis was performed. As depicted in Fig. 4b the number of TH neurons was significantly decreased in P-PFF injected animals (76,8 ± 3,2%, percentage ipsi/contra). No significant differences were observed between the ipsi- and the contralateral side of the other fibrilar types or the PBS control (wt-PFF:100 ± 3,8% vs S129A-PFF:98,4 ± 7,2% vs PBS:98,5 ± 4,4% percentage ipsi/contra). As expected, no differences were observed in P-PFF injected α-Synuclein null mice (98,1 ± 2,6%, percentage ipsi/contra) (Fig. 4b). Similar to TH, P-PFF injected animals exhibited a significant loss of VMAT2 staining between the contra and the ipsilateral side (Fig. 4c), (TH (contra:5793 ± 385 vs ipsi: 4501 ± 466), and VMAT2 (contra :5446 ± 505 vs ipsi: 4158 ± 403) numbers of dopaminergic neurons). In agreement with a dopaminergic neuronal loss in the SNpc, HPLC analysis also revealed significant reductions in the striatal concentrations of dopamine (DA) in the P-PFF-injected side (P-PFF:0,59 ± 0,05 vs wt-PFF:0,88 ± 0,04 vs S129A-PFF:0,83 ± 0,07 vs PBS: 0,86 ± 0,04 ratio ipsi/contra). DA levels were not affected in the α-Synuclein null P-PFF injected animals (0,98 ± 0,07 ratio ipsi/contra) (Fig. 4d). These data further suggest that the differential effect of phosphorylated fibrils depends on the seeding of endogenous α-Synuclein, since P-PFF injections in α-Synuclein null mice did not affect dopaminergic neuron integrity. Given the SNpc neuron loss, and reduced striatal DA levels, PFF-inoculated wt mice were examined for motor function. To this end, locomotor activity was assessed in an open field. No difference in distance traveled between P-, wt-, and S129A-PFF injected mice vs. control PBS was observed. α-Synuclein null mice injected with the three different fibrilar types also did not show differences in locomotor activity (Supplementary Fig. S5a). Challenging beam traversal was used to assess fine motor function. A statistically significant increase in errors/step in P- vs. S129A-PFF and PBS-injected mice was observed (P-PFF:0,5 ± 0,04 vs wt-PFF:0,42 ± 0,04 vs S129A-PFF:0,35 ± 0,01 vs PBS: 0,35 ± 0,02) (Fig. 4e). No changes were observed in wt-, P-, S129A-PFF and PBS-injected α-Synuclein null mice (Fig. 4e). No significant differences were observed in the rotarod, pole test and grip strength (Supplementary Fig. S5b,c,d).

### Phosphorylated-PFF induce robust seeding of α-Synuclein pathology in cortical regions

**Figure 5:**
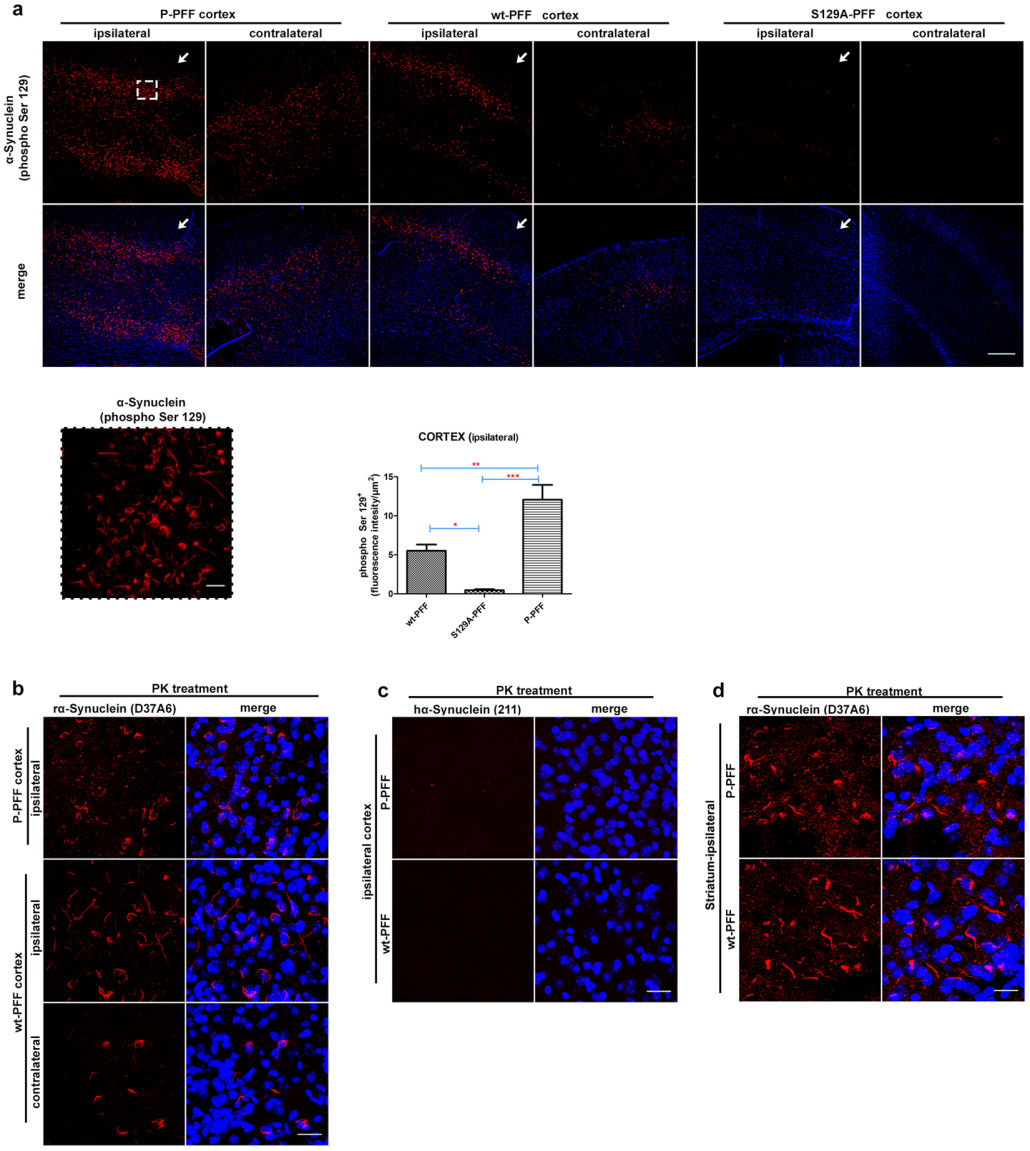
Robust endogenous α-Synuclein accumulation is also evident in the cortex of injected animals. Coronal sections of P-, wt- and S129A- PFF injected animals were stained for α-Synuclein (phospho Ser129). (**a**) P-α-Synuclein positive inclusions (highlighted in magnification within the dashed line frame) were evident in the ipsilateral cortex of P- and wt- PFF-injected animals. Progression of pathology but in a more dispersed pattern is also evident in the contralateral cortex. S129A- PFF are not as efficient in inducing α-Synuclein accumulation on either sides. Arrows indicate the needle entry point. Graph shows phospho Ser129 mean fluorescence intensity of the ipsilateral cortex, normalized to the measured area (intensity/μm^2^) (n = 4 animals/group, p = 0,0002). (**b**) The rodent specific anti-α-Synuclein (D37A6) antibody and (**c**) the human specific anti-α-Synuclein (211) were used for immunostaining of PK-treated sections (10 min at 25 °C to expose the antigenic sites). Pathological accumulations in the area of the cortex of both hemispheres were positively stained with the rodent specific antibody. α-Synuclein accumulations did not stain with the human specific anti-α-Synuclein (211) antibody in the ipsilateral cortex of P- and wt-PFF injected animals (n = 4). (**d**) Coronal striatal sections of P- and wt-PFF injected animals were also positively stained with the rodent specific α-Synuclein (D37A6) antibody. TO-PRO-3 (blue) was used as a cell nuclear marker. Scale bars represent 250 μm in (**a**) and 25 μm in (**b,c,d**).

The dorsal striatum receives various direct and indirect combinations of different inputs from multiple brain regions^17^. Robust P-α-Synuclein positive inclusions were evident in the ipsilateral cortex of injected animals, in different layers. Interestingly, neurons in the contralateral cortex also accumulated inclusions, but in a more disperse pattern (Fig. 5a). Similar to our findings in the SNpc (Fig. 4a), fluorescence intensity quantification of the α-Synuclein (phospho S129) in the tiled cortical region (see materials and methods) revealed that all three fibrilar types were able to induce pathological accumulations in the cortex. However, there is a significant difference as to the seeding capacity of the different types. As shown in Fig. 5a, P-PFF are more potent to induce pathological accumulation of α-Synuclein in other interconnected brain regions and to hasten pathology in regions of the contralateral side 60 dpi (ipsilateral cortex: P-PFF:12,06 ± 1,8 vs wt-PFF:5,52 ± 0,79 vs S129A-PFF:0,47 ± 0,1, fluorescence intensity/μm^2^ cortical tissue). To further prove that the observed induction of pathology results from a progressive accumulation of the endogenous α-Synuclein in both hemispheres and not by diffusion of the injected material we stained the sections with antibodies specific to α-Synuclein species. Following PK treatment the sections were stained with the rodent specific D37A6 antibody as well as the human specific 211. Pathological accumulations in the area of the cortex of both hemispheres were positively stained only with the rodent specific antibody (Fig. 5b,c). Extensive PK treatment allowed for the detection of PK resistant D37A6 positive accumulations in cortical regions of the injected animals (Supplementary Fig. S6). Rodent α-Synuclein accumulation was also observed in the ipsilateral striatum of injected animals (Fig. 5d).

### PFF-induced α-Synuclein inclusions in the CNS are SDS-resistant

**Figure 6:**
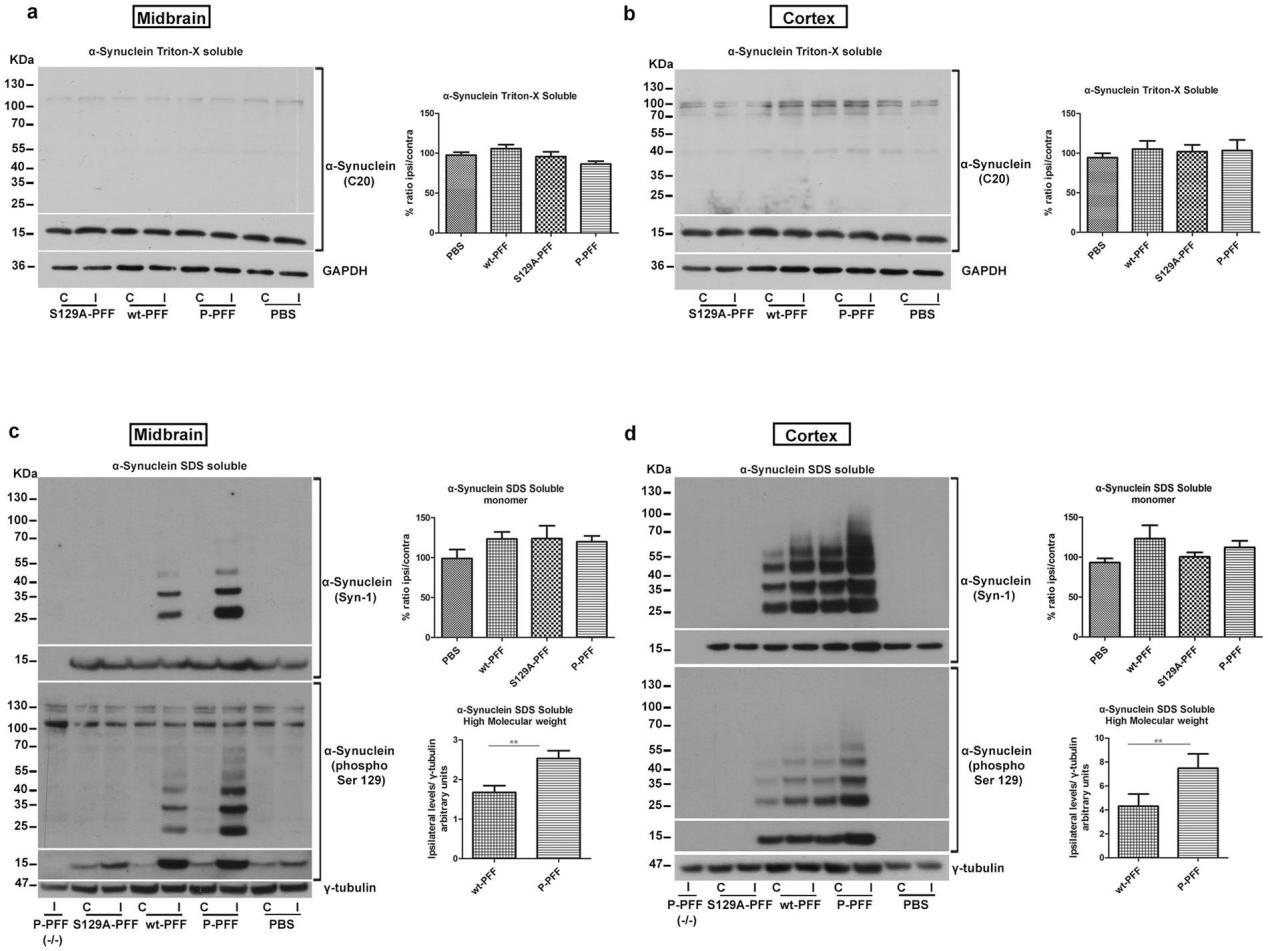
Biochemical profile of α-Synuclein in PFF-injected animals. (**a**) Midbrain Triton-X soluble samples of injected animals showed no difference in α-Synuclein levels between the ipsi-and contralateral side in all fibrilar types and the control PBS- injected animals (α-Synuclein monomer is shown in cropped gel/blot). GAPDH was used as a loading control (cropped gel/blot is shown) (n = 5–7 brains/group). Similar in (**b**) no differences were found for the Triton-X soluble fraction in the area of the cortex (α-Synuclein monomer is shown in cropped gel/blot) (n = 4 brains/group). (**c**) P- and wt-PFF injections resulted in a shift of the SDS-soluble α-Synuclein in higher molecular weight species ipsilaterally in the midbrain. These SDS-soluble α-Synuclein fraction was significantly enriched in the P-PFF injected side compared to the wt-PFF treatment (n = 5 brains/group). High molecular weight species in both treatments were also positive for the phospho Ser 129 α-Synuclein antibody. No difference was observed in the SDS-soluble α-Synuclein monomer levels between the ipsi- and the contralateral side for the P-, wt-, S129A-PFF and the PBS-injected animals (α-Synuclein monomer is shown in cropped gel/blot) (n-6–7 animals/group). γ-tubulin was used as a loading control (cropped gel/blot is shown). (**d**) Immunoblot for the SDS-soluble fraction extracted from the cortex of injected animals showed that α-Synuclein SDS-soluble high molecular weight species are formed readily in P-PFF-injected animals in both sides compared to the wt treatment. Densitomentry of the ipsilateral α-Synuclein levels confirmed the significant difference between the treatments (n = 5 animals/group). α-Synuclein null mice (−/−) injected with P-PFF did not show any positive signal with the Syn-1 antibody. The observed α-Synuclein species were phosphorylated in nature as seen following immunoblotting with the phospho Ser 129 antibody. No difference was observed in the SDS-soluble α-Synuclein monomer levels between the ipsi- and the contralateral side for all types of injected animals (α-Synuclein monomer is shown in cropped gel/blot) (n = 5–6 animals/group). γ-tubulin was used as a loading control (cropped gel/blot is shown). Data represent mean values ± SEM. Differences were estimated using one-way ANOVA followed by Tukey’s post-hoc test and paired two-tailed Student’s t-test. (**c**) p = 0,0037 (**d**) p = 0,0053.

To further assess the biochemical profile of α-Synuclein in animals injected with the three different PFF types we also performed sequential protein extraction of the ventral midbrain and the cortex. Western blotting of the Triton X-soluble fraction with the C20 antibody did not reveal any significant differences in α-Synuclein levels between the two hemispheres in both regions (Fig. 6a,b). However, as shown by western blotting with the Syn-1 antibody, α-Synuclein in the SDS-soluble fraction of the ventral midbrain migrated at higher molecular weights in the injected side of both P- and wt-PFF treated animals. P-PFF treatment caused a statistical significant increase in α-Synuclein species of high molecular weights compared to wt-PFF (P-PFF: 2,53 ± 0,19 vs wt-PFF:1,67 ± 0,17 ipsilateral levels normalized to γ-tubulin) (Fig. 6c, top panel & graph). As expected, these high molecular weight SDS-soluble species were phosphorylated at S129 (Fig. 6c, lower panel). No changes were observed in monomeric, SDS-soluble, α-Synuclein levels (Fig. 6c & graph). We did not observe any monomeric or high molecular weight α-Synuclein species in α-Synuclein null mice injected with either wt-PFF or P-PFF. Similar analysis was performed in the cortex of the injected mice. Consistent with the severity of pathology as it is also depicted in Fig. 5a, α-Synuclein in the SDS-soluble fraction migrated at higher molecular weights in the cortex of animals injected with P- and wt-PFF both ipsi- and contralaterally. A significant increase in high molecular weight α-Synuclein species was observed in the cortex of animals injected with P-PFF (P-PFF: 7,48 ± 1,2 vs wt-PFF:4,31 ± 1, ipsilateral levels normalized to γ-tubulin) (Fig. 6d, top panel & graph). No changes were observed in the monomeric, SDS-soluble, α-Synuclein levels (Fig. 6d & graph). Again, monomeric or high molecular weight α-Synuclein species were not present in null mice injected with either wt-PFF or P-PFF. As expected, cortical P-α-Synuclein species were abundant in both the ipsi-and contralateral side of P-PFF and wt-PFF-injected animals (Fig. 6d, lower panel). High molecular weight α-Synuclein species were absent in PBS-injected animals (Fig. 6d).

### PFF injected mice exhibit different innate immune cell responses in the CNS

**Figure 7:**
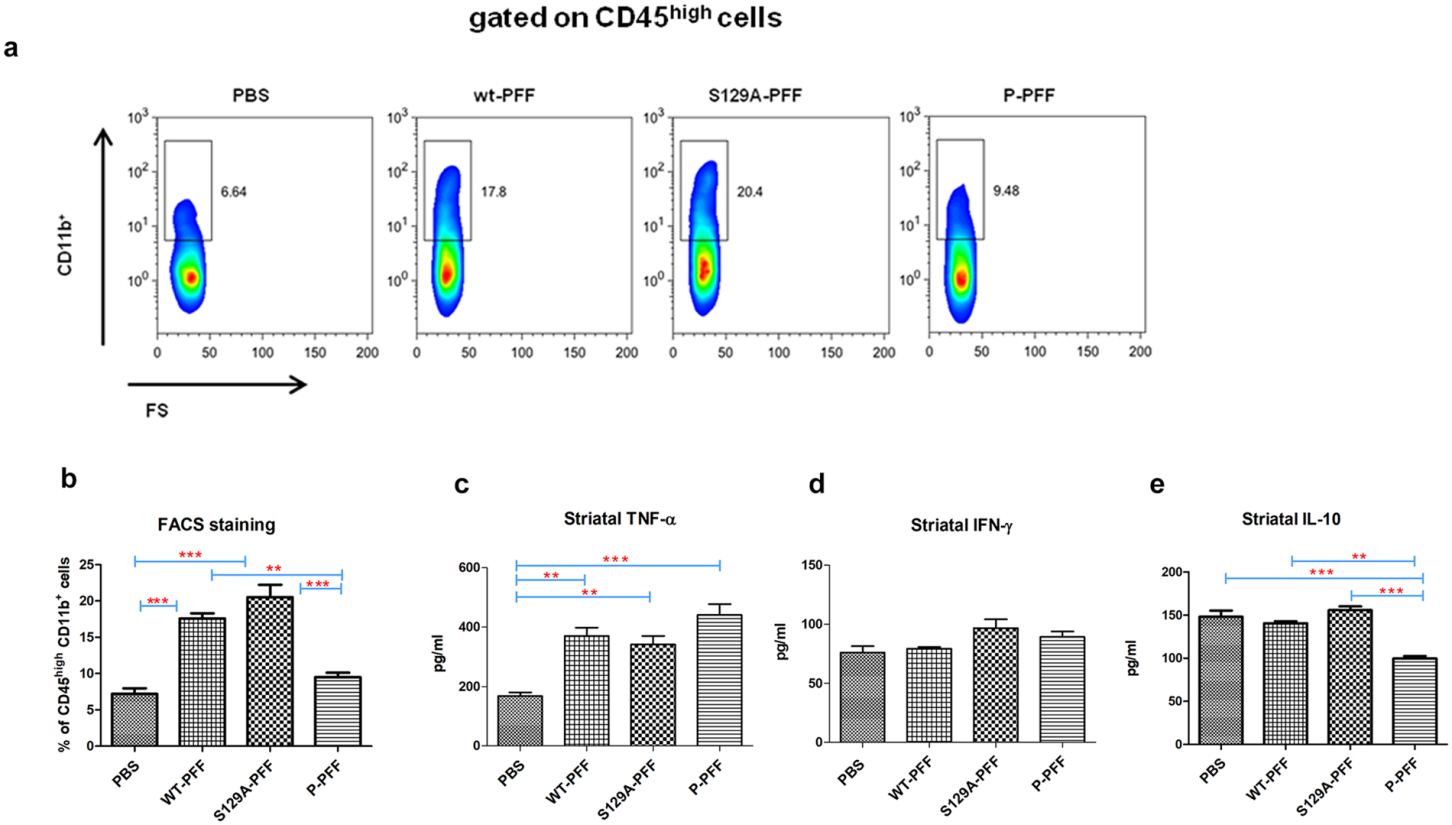
Reduced innate immune response following P-PFF treatments *in vivo*. (**a**) Cells obtained from ipsilateral striatal tissue of 3 dpi injected mice were stained and analyzed by flow-cytometry. Each animal was analyzed individually. Representative FACS plots showing the percentages of CD11b^+^ cells gated on CD45^high^ leukocytes. (**b**) Cumulative data showing the percentages of CD45^high^CD11b^+^ macrophages in injected animals. Data are pooled from three independent experiments. (**c**) TNF-α (**d**) INF-γ and (**e**) IL-10 levels were measured in ipsilateral striatum homogenates by ELISA. Data are expressed as mean ± SEM of triplicate wells. Data shown for cytokine release are pooled from three independent experiments. Statistical significance was obtained by ANOVA followed by Tukey’s post-hoc test. (**b**) p < 0,0001 (**c**) p = 0,0007 (**e**) p = 0,0001.

It is possible that differences in the phosphorylation state of fibrils may lead to differences in their uptake or clearance by immune cells, such as macrophages and/or microglia^18^. This could be irrelevant to their potency to seed de novo misfolding of native α-Synuclein within neurons. We therefore investigated the effects of the three different fibrilar types on the activation of immune responses in the brains of injected mice. We focused on the examination of innate cell responses in the striatum induced early during the course of inclusion formation (3 dpi). Flow cytometry analysis of distinct innate immune cell types in the striatum demonstrated that mice injected with wt- and S129A-PFF exhibited enhanced frequencies of infiltrating peripheral macrophages, identified as CD45^high^CD11b^+^ at the ipsilateral side (Fig. 7a), (gating strategy shown in Supplementary Fig. S7a)^19^. In contrast, mice injected with P-PFF had markedly decreased percentages of CD45^high^CD11b^+^ macrophages (PBS:7,2 ± 0,7% vs wt-PFF:17,6 ± 0,6% vs S129A-PFF:20,53 ± 1,7% vs P-PFF:9,5 ± 0,6%) (Fig. 7a,b). Examination of CD45^low^CD11b^+^ cells, generally considered as resident microglia cells^19^, did not show significant differences in their frequencies among the groups studied (Supplementary Fig. S7b). Given the marked differences observed in the percentages of infiltrating macrophages, we next investigated their immunophenotype. No differences were observed in the expression of the macrophage activation markers, MHC-II and CD86, at the cell surface (Supplementary Fig. S8a and b). Interestingly, mice injected with P-PFF exhibited higher percentages of NOS2^+^-expressing CD45^high^CD11b^+^ cells, resembling pro-inflammatory M1-like macrophages^18^, as compared to the other groups (P-PFF:29,2 ± 1,7% vs PBS:14,5 ± 0,9% vs wt-PFF:15,5 ± 1,1% vs S129A-PFF:15,1 ± 1,2%), while no differences were observed in the frequencies of Arg1^+^-expressing CD45^high^CD11b^+^ cells, considered anti-inflammatory M2-like macrophages (Supplementary Fig. S9a and b). Examination of the profile of cytokines released in the striatum showed that mice injected with all PFF types had significantly increased TNF-α levels compared to PBS-injected controls (PBS:167,7 ± 12,7 vs wt-PFF:369,7 ± 28,17 vs S129A-PFF:341,3 ± 28,6 vs P-PFF:440,8 ± 36,5) (Fig. 7c), while IFN-γ levels were similar in all groups (Fig. 7d). IL-6 was undetectable in all groups studied (data not shown). In contrast, IL-10 levels were significantly decreased in mice injected with P-PFF, compared to those administered PBS, wt- or S129A-PFF (PBS:148,1 ± 7,2 vs wt-PFF:140,5 ± 2,4 vs S129A-PFF:155,7 ± 4,3 vs P-PFF:99,8 ± 2,7) (Fig. 7e). To determine the cell source of IL-10 in the striatum, we performed flow cytometry analysis in WT- and P-PFF injected mice. Both resident CD45^low^CD11b^+^ microglia and infiltrating CD45^high^CD11b^+^ macrophages expressed IL-10 in wt-PFF injected mice. Still, mice injected with P-PFF exhibited decreased frequencies of IL-10-producing macrophages and microglia (Supplementary Fig. S10a and b). Investigation of cytokine levels at the later time point of 60 dpi, demonstrated that TNF-α levels remained significantly elevated in the striatum in the P-PFF compared to PBS-injected animals (P-PFF:1281 ± 56,41 vs PBS:1013 ± 29,81 vs wt-PFF:1061 ± 75,48 vs S129A-PFF:1042 ± 72,84), while IL-10 and IFN-γ were similar in all groups studied (Supplementary Fig. S11a and b).

### Increased uptake of P-PFF by primary neurons

**Figure 8:**
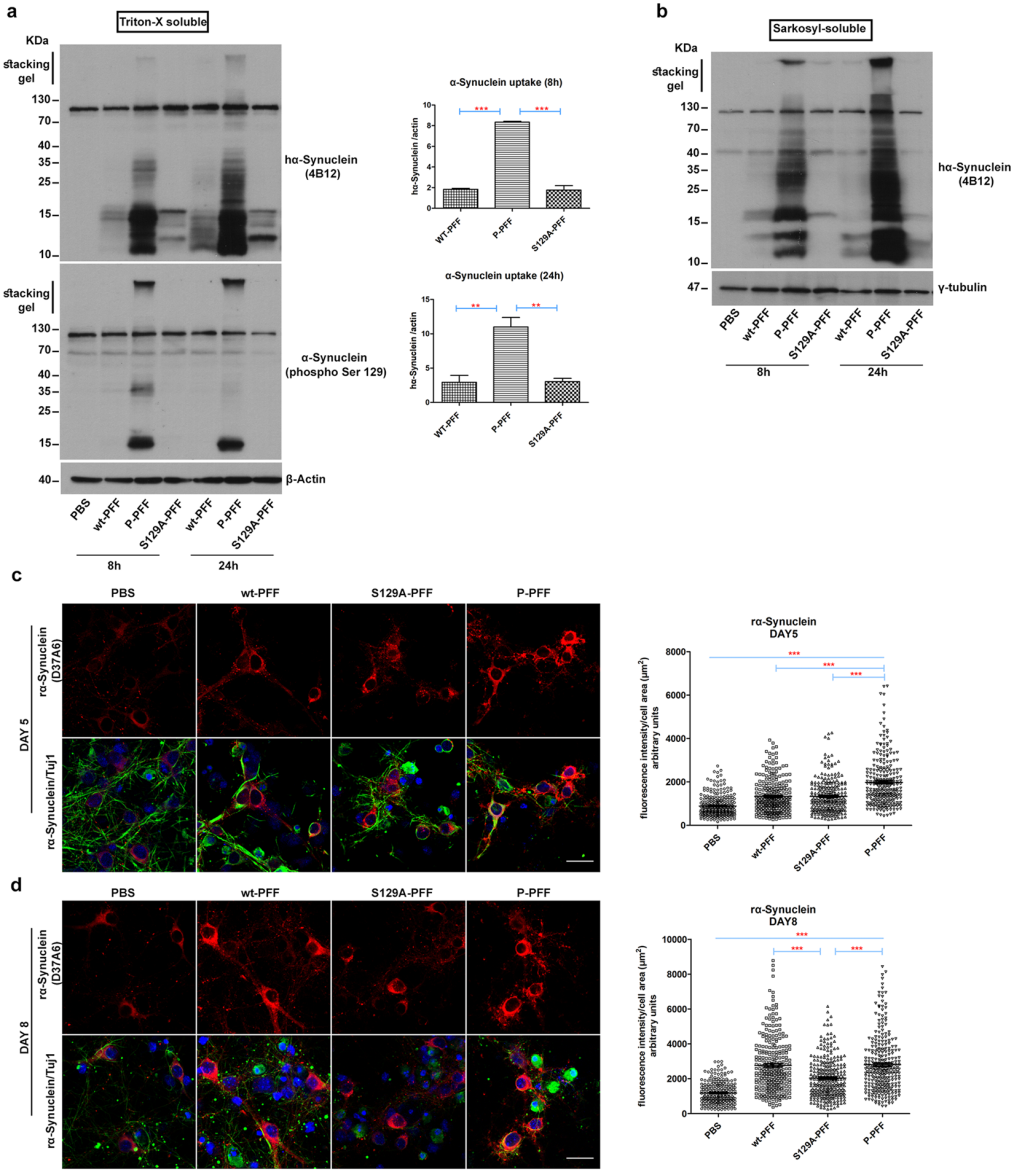
Increased uptake and faster seeding of the endogenous α-Synuclein in primary neurons following P-PFF treatment (**a**) Mouse primary cultures (6div) were treated with PFF for 8 and 24 hours. Following Triton-X extraction, internalized fibrils were visualized with the human specific α-Synuclein antibody 4B12. Increased uptake of P-PFF was observed as early as 8 h. The uptake was quantified by densitometry and found to be significantly increased for P-PFF in both time points compared to wt- or S129A- PFF. P-PFF uptake was further verified using the phospho Ser 129 antibody. β-Actin was used as a loading control (cropped gel/blot is shown). (**b**) Sarcosyl-soluble α-Synuclein species were also increased following P-PFF treatment of primary cortical neurons. γ-tubulin was used as a loading control (cropped gel/blot is shown). (**c**) Immunocytochemistry with the rodent specific α-Synuclein antibody (D37A6) in primary mouse cortical cultures treated with the three fibrilar types. P-PFF seed the endogenous α-Synuclein more effectively compared to the other fibrilar types and PBS control treated neurons for 5 days. However (**d**) 8-day-treated neurons exhibit similar levels of endogenous α-Synuclein signal for both the wt- and P-PFF treatments, in contrast to the significant lower levels of S129A-treated neurons. Scatter plots present the mean fluorescence intensity/cell of three independent experiments (n≈90 single cells/condition per replicate). β Tubulin III (Tuj 1) was used as a neuronal marker. TO-PRO-3 (blue) was used as a cell nuclear marker. All data represent mean values ± SEM from three independent experiments. Statistical significance was obtained by ANOVA followed by Tukey’s post-hoc test for (**a**) and non parametric Kruskal-Wallis test followed by Dunn’s post-hoc test for (**c** and **d**). (**a**) For 8 h p < 0,0001 and for 24 h p = 0,002, (**c**) p < 0,0001 (**d**) p < 0,0001.Scale bar in (**c** and **d**) represent 25 μm.

To better understand why P-PFF induce faster accumulation of P-α-Synuclein pathology following intrastriatal injections we incubated wt-, P- and S129A-PFF with mouse primary cortical cultures for 8 and 24 hours (see Materials and Methods). Following trypsinization, cell lysates were sequentially fractioned with Triton-X, and Sarcosyl. Internalized α-Synuclein species were detected in the fractions by immunoblotting with the human specific α-Synuclein antibody 4B12. As shown in Fig. 8a, all three fibrilar types are internalized by neuronal cells. However, compared to wt- or S129A-PFF, P-PFF exhibit increased intracellular accumulation evident as early as 8 h following incubation with primary neuronal cultures (Fig. 8a). The fast uptake of the P-PFF, was further verified by immunoblotting with the α-Synuclein (phospho Ser129) specific antibody (Fig. 8a). Quantification of internalized human α-Synuclein species revealed a significant increase in P-PFF uptake at 8 h and 24 h following incubation with primary neuronal cultures (for 8 h, wt-PFF:1,84 ± 0,1 vs P-PFF:8,34 ± 0,1 vs S129A-PFF:1,78 ± 0,42 and for the 24 h, wt-PFF:2,95 ± 0,99 vs P-PFF:11,01 ± 1,36 vs S129A-PFF:3,03 ± 0,47). Similar results were obtained when probing the Sarkosyl soluble (Fig. 8b) and the insoluble fractions with the 4B12 antibody (Supplementary Fig. S12a). Interestingly, we observed additional α-Synuclein cleavage products in both the Triton-X and Sarcosyl fractions which could be the result of frank cleavage of the fibrils. To investigate whether the uptake of P-PFF by neuronal cultures was dose dependent, we incubated cells with half the amount of P-PFF. As shown in Supplementary Fig. 12b reducing the amount of P-PFF did not affect their ability to be uptaken by neuronal cells and still demonstrated increased internalization compared to the higher doses of wt- and S129A- PFF.

To examine the effect of the observed increased uptake on the seeding of endogenous α-Synuclein, we treated primary cortical cultures with the three types of fibrils for 5 and 8 days. Incubation of cortical neurons with all three fibrilar types for 5 days resulted in foci formations, originating from the endogenous α-Synuclein accumulation (Supplementary Fig. S12c **)**. Endogenous α-Synuclein was monitored using a rodent specific antibody for 5 and 8 days. As depicted in Fig. 8c, treatment with all PFF types caused a significant increase in the mean fluorescence intensity/cell as early as 5 days post-treatment compared to control PBS-treated cultures (PBS:894,1 ± 29,7 vs wt-PFF:1337 ± 47,9 vs S129A-PFF:1337 ± 48,5 vs P-PFF:1980 ± 69,1 mean fluorescence intensity/cell area). However, the endogenous mean fluorescence signal in P-PFF-treated neurons was estimated to be approximately 48% more compared to that of wt or mutant S129A-treated cells which exhibited comparable levels of fluorescence intensity (Fig. 8c). On day 8, cells treated with the wt- or P-PFF exhibited similar levels of mean fluorescence signal intensity (PBS: 1185 ± 41,5 vs wt-PFF:2767 ± 94,9 vs S129A-PFF:2036 ± 67,7 vs P-PFF:2806 ± 93,5 mean fluorescence intensity/cell area) (Fig. 8d). In contrast, the mean fluorescence intensity of mutant S129A-treated neurons was found significantly lower compared to the wt-or P-PFF treatment but still significantly increased versus control PBS-treated cells (Fig. 8d). To assure that the effects of our PFF preparations were not due to the phosphorylation procedure followed, mutant S129A-PFF were phosphorylated *in vitro* by PLK2, (mock-P) S129A-PFF. Notably, in both phosphorylated fibrilar preparations we were not able to detect any PLK2 positive signal by western blot analysis (Supplementary Fig. 13a). Primary cortical neurons were treated for 24 h with either S129A-PFF or its phosphorylated counterpart (mock-P) S129A-PFF and P-PFF as described previously. Immunoblotting of the Triton-X soluble fraction with the human specific α-Synuclein antibody 4B12 showed that S129A-PFF and (mock-P) S129A-PFF were uptaken by the cells in a similar pattern. As expected, both types exhibited decreased intracellular accumulation compared to P-PFF (Supplementary Fig. 13b). Furthermore, incubation of cortical neurons with S129A-PFF and (mock-P) S129A-PFF for 5 days exhibited comparable levels of endogenous α-Synuclein accumulation monitored by the fluorescence intensity of the rodent specific antibody D37A6. Both treatments resulted in increased endogenous signal compared to control PBS-treated neurons, but still significantly lower compared to P-PFF treated neurons (PBS: 1317 ± 43,08 vs S129A-PFF:1940 ± 88,74 vs (mock-P) S129A-PFF:1845 ± 72,86 vs P-PFF:3038 ± 119,3 mean fluorescence intensity/cell area) (Supplementary Fig. S13c).

## Discussion

Various *in vivo* studies that have investigated the effects of S129 phosphorylation of α-Synuclein have failed to reach a consensus as to the function of this modification. To gain insight into the role of phosphorylation in templating the pathology of α-Synuclein *in vivo*, we generated phosphorylated recombinant human-α-Synuclein fibrils (P-PFF) and we performed stereotaxic injections targeting the dorsal striatum of wt and α-Synuclein null mice. In comparison, mice were inoculated with wt-PFF and mutant S129A-PFF that bear the substitution Serine to Alanine and are incapable of *de novo* phosphorylation at this site. It has been shown in a variety of animal models of induced pathology by misfolded α-Synuclein that regardless of the nature of the injected material (i.e. pre-formed α-Synuclein fibrils, extracts from human brains with Dementia with Lewy Bodies (DLB) or Parkinson’s disease and brain lysates from symptomatic α-Synuclein overexpressing mice) and irrespective of the initial site of injection, a robust α-Synuclein pathology could be detected away from the injection site^14,15,20–22^. To evaluate the effects of the different fibrilar types in brain pathology progression we chose the early time point of 60 dpi for our analyses. Given the fact that the dorsal striatum receives its main afferents from the dopaminergic neurons of the SNpc^23^ which are susceptible in Parkinson’s disease, we first examined whether all of the three types of injected fibrilar α-Synuclein could affect healthy neurons in interconnected areas away from the injection site in accordance with the recently proposed theory of pathology transmission to explain neurodegenerative disease progression^24–26^. Inoculation of all three human fibrilar α-Synuclein types in wt mouse brain showed pathologic accumulations by incorporation of endogenous α-Synuclein. These aggregates were also recognized by conformation specific oligomeric antibodies to α-Synuclein. Our results are in agreement with those of Masuda and co-workers^21^, who showed that recombinant human fibrilar α-Synuclein or α-Synuclein extracted from human DLB brains was efficient to induce pathology in mice albeit at much later time points. The types of PFF used in our study, induced histopathological and neurotoxic features that appear dependent on the post-translational modification (PTM) properties of the fibril. In a recent report Osterberg and colleagues^27^ demonstrated using *in vivo* imaging a stage-like, progressive maturation of inclusions following PFF inoculation. In this sense, the three fibrilar types used in our study could be generating pathology of different maturities along the same continuum, rather than distinct pathologies. The structural differences between the P-PFF and the wt-PFF, in our view, are minor since the first are produced by the *in vitro* phosphorylation of the wt-PFF. On the other hand we cannot exclude the possibility that the mutant S129A-PFF might form different fibrilar species which are less potent in inducing robust pathology at the given time point. It is therefore, tempting to assume that phosphorylation of filamentous forms of α-Synuclein, is critical for the acceleration of pathogenesis. This notion was further strengthened by the observation that unilateral P-PFF injections induced a more severe phenotype of α-Synuclein pathology in the contralateral side. In our study, there was no obvious difference in PK resistance between the phosphorylated and non-phosphorylated forms. It is feasible that structural differences and differences in phosphorylation may lead to enhanced uptake or clearance of fibrils that is irrelevant of their potency to seed de novo misfolding of native α-Synuclein within neurons. Our *in vitro* experiments demonstrated that the exacerbated pathology observed following P-PPF brain inoculation could be attributed partly to increased uptake of the P-PFF and thus faster access to the cytosolic α-Synuclein. In this sense, P-PFF appear to have an increased capacity to seed the endogenous α-Synuclein compared to wt-PFF. Even when used at a lower concentration, P-PFF retained their ability for faster uptake by neurons. Interestingly, the S129 phosphorylation-incompetent fibrils exhibit reduced uptake by primary neurons and a slower ability in seeding cytosolic α-Synuclein. In agreement with recent reports, exogenous α-Synuclein fibrils efficiently attached to cell membranes and were subsequently internalized^28^. Similarly, Filsy *et al*., recently demonstrated that S129 phosphorylation slightly increased membrane binding and cellular uptake of P-PFF *in vitro* ^29^. Different approaches to generate phosphorylated α-Synuclein fibrils have been used in the literature varying mainly in the kinases and the phosphorylation approach used (direct *in vitro* phosphorylation, co-expression of α-Synuclein with PLK2 in *E.coli* and chemical synthesis) with equivocal results in fibrillization kinetics and properties of the resulted fibrils^3,8,29–31^. Here we chose to first aggregate and then phosphorylate the mature wt-PFF in order to minimize the interference of phosphorylation in the fibrillization process. The effective phosphorylation was confirmed with specific commercial and “in house” newly designed antibodies and LC-MS/MS analysis (Supplementary Fig. S1 and S2). Whether phosphorylated fibrils bind the newly identified LAG3 receptor^32^ with higher affinity also remains to be investigated. It is noteworthy, that the injected fibrils in our study did not appear to change their PTM profile following intrastriatal injection (Fig. 2c). Interestingly, enhancement of the activity of protein phosphatase 2 A (PP2A) in wild-type α-Synuclein transgenic mice improved motor impairments, increased neuronal activity and dendritic arborization^33^. Our results using the phosphorylation-incompetent S129A-PFF are in agreement with data from Luk *et al*.^15^, as we show that although phosphorylation is not required for the formation of pathologic inclusions, it appears to produce a more severe pathology. Still, host expression of soluble α-Synuclein is an absolute prerequisite for the *de novo* formation of these inclusions because inoculation of the P-PFF in α-Synuclein null animals, which express neither mouse nor human α-Synuclein, resulted in no P-α-Synuclein immunostaining at 60 dpi.

Importantly, and in the early time point used in our study (60 dpi), the nigra displayed significant α-Synuclein accumulation in the absence of neuronal loss in the S129A-and wt-PFF injected animals. However, our experiments demonstrate that P-PFF induce acute neuronal loss. In this respect, DA levels were found significantly reduced only in P-PPF injected animals and motor-coordination was compromised within 60 dpi. Challenging beam traversal was included for the first time to behaviorally assess α-Synuclein fibril-injected mice. P-PFF injected mice performed worse in beam traversal, indicating impaired motor coordination without affecting general locomotor function. Perhaps the lack of a similar motor deficit in pole descent or rotarod performance could be explained by the nature of these tests that may more easily allow for compensation in the presence of motor asymmetry.

Tissue fractionation revealed that in P-PFF treated animals a higher amount of α-Synuclein was recovered in the SDS-soluble fraction compared to wt- or S129A-PFF treatments especially in the cortex. These aggregated species were also found to be heavily phosphorylated and matched the pattern of the severity of pathology in the brain produced by wt- and P-PFF seen in our immunohistochemical analysis. These data collectively suggest that the α-Synuclein species formed following P-PFF-treatment are highly aggregated.

Interestingly, our studies revealed an enhanced recruitment of macrophages from peripheral lymphoid compartments into the striatum, in response to injection with wt- and S129A-PFF, while mice injected with P-PFF exhibited markedly decreased macrophage infiltrations. A growing body of evidence suggests that activation of innate immune responses mediated by peripheral macrophages and microglia, early during the course of CNS inflammation, represents an inherent protective mechanism as it facilitates the clearance of pathogenic α-Synuclein deposits/accumulations and suppresses the generation of detrimental neuroinflammatory processes involved in neurodegeneration^34^. Hence, it is conceivable that decreased macrophage recruitment and/or activation in the CNS in mice injected with P-PFF may, at least, partly, contribute to the enhanced a-Synuclein accumulation and the exacerbated pathology in these mice. In support, the levels of the anti-inflammatory cytokine IL-10, produced also by infiltrating macrophages as shown in the present study, were significantly decreased in the CNS of mice injected with P-PFF, further emphasizing a decreased capacity to initiate protective immunosuppressive mechanisms. In contrast, TNF-α levels were significantly increased, both early and later during the course of inclusion formation, in mice injected with P-PFF, highlighting increased inflammatory responses in the CNS of these mice. In agreement with our studies, *Lee et. al*. have demonstrated that *in vivo* dephosphorylation of S129 led to fewer α-Synuclein aggregates and reduced inflammation in α-Synuclein overexpressing mice^33^. Taken together, it seems that different types of fibrils activate innate immune responses in a distinct manner, with P-PFF failing to mount a protective immune cell-mediated response. Moreover, *in vivo* studies by *Christiansen et. al*. have demonstrated that vaccination with distinct doses of α-Synuclein and/or with nitrated α-Synuclein induces the expansion and recruitment of different T effector and T regulatory cell types in the CNS^35^. Overall, our data highlight the importance of phosphorylation in the early events of α-Synuclein pathology. Our experiments suggest that phosphorylation, which appears to be critical in Parkinson’s disease, adds “aggregation” capacity to the α-Synuclein assemblies and as such may regulate the onset of neuronal dysfunction.

## Materials and Methods

### Animals

Equal numbers of male and female wild-type C57BI6/C3H mice 2–4 month old (Jackson Laboratory, Bar Harbor, Main) and α-Synuclein null mice (C57BL6/JOlaHsd mice, Harlan Laboratories) were used. Depending on the experiment, a minimum of 3 or 5 animals were used per condition and per treatment. Animals were housed in the animal facility of the Biomedical Research Foundation of the Academy of Athens in a room with a controlled light-dark cycle (12 h light–12 h dark) and free access to food and water.

### Expression and purification of recombinant human wt α-Synuclein

The expression and purification of α-Synuclein has been described elsewhere^16,36,37^. Briefly, the GST-α-Synuclein fusion construct in pGEX-4T1 vector was expressed in *E.coli* BL21 bacteria. The supernatant was purified with affinity chromatography using glutathione sepharose 4B beads (Amersham, Sweden), and the GST-α-Synuclein bound to beads was cleaved by thrombin. α-Synuclein was collected by centrifugation and purified after GST tag cleavage using reversed phase HPLC. The purified eluted protein was dialyzed in 1x PBS and the final concentration was estimated by BCA assay according to the manufacturer’s instructions (Thermo Fisher Scientific,USA).

### Expression and purification of recombinant human S129A α-Synuclein

pT7–7 α-Synuclein S129A was a gift from Hilal Lashuel (Addgene, plasmid # 36048)^8^. The expression vector was transformed into *E-Coli* BL-21 bacteria. The bacterial lysate was injected into Superdex 200 gel filtration column, followed by anion exchange purification by Mono-Q column. The S129A α-Synuclein monomer purity was confirmed by commassie blue staining with different amounts of the protein (Supplementary Fig. S14a). The purified protein was then dialyzed in 1x PBS and the final concentration was estimated by BCA assay according to the manufacturer’s instructions (Thermo Fisher Scientific,USA).

### Aggregation of α-Synuclein *in vitro* (wt-PFF and S129A-PFF)

The samples of 100 µM α-Synuclein were incubated at 37 °C for 7 days with continuous shaking at 800 rpm in a Thermomixer (Eppendorf, Germany). Samples were collected at indicated time points and Thioflavin-S assay was carried out to monitor the fibril formation (see Supplementary Materials and Methods). The resulting fibrils were then centrifuged for 10 minutes at 18,000 g to collect the insoluble fibrils and subjected to sonication.

### *In Vitro* phosphorylation of α-Synuclein PFF (P-PFF)

The wt-PFF sample was fragmented by ultra-sonication on ice using Sonic ruptor 250, equipped with a fine tip (5-s pulses, output of 40 watts for 5 min). The sonicated PFF (100 μM) then were phosphorylated using polo-like kinase 2 (PLK2) (Invitrogen, Thermo Fisher Scientific, USA), as previously described^38^. The reaction was carried out in a final volume of 100 μl. The 1x phosphorylation buffer (20 mM HEPES, 1,09 mM ATP, 2 mM DTT, 10 mM MgCl2) and 1 μg PLK2/144 μg of fibrils were incubated overnight at 30 °C. The fibrils were washed 2 times with ultra-pure water and resuspended in 1x PBS and stored at −80 °C. Phosphorylation of monomeric wt α-Synuclein was monitored in different time points (Supplementary Fig. S14b). To assure that the effects of our PFF preparations were not due to the phosphorylation procedure followed, we also subjected the mutant S129A-PFF to *in vitro* phosphorylation by PLK2 as described above. To validate the specific phosphorylation of P-PFF at S129 position, the α-Synuclein C-terminal fragment was isolated by AspN digestion and LC-MS/MS analysis was performed (For detailed protocol see Supplementary Materials and Methods).

### Transmission Electron Microscopy (TEM)

Electron microscopy images were produced by adding 5 µl of the sample on Formvar-coated 400 mesh copper grids, fixed briefly with 0.5% glutaraldehyde (5 µl), negatively stained with 2% uranyl acetate (Sigma-Aldrich, USA) and examined in a Philips CM-10 TEM electron microscope.

### Stereotaxic injections

Unilateral striatal injections were performed under general isoflurane anesthesia by an apparatus adjusted to the stereotaxic frame (Kopf Instruments, USA). Right dorsal striatum was targeted using the following coordinates from bregma: anteriorposterior +0,5 mm, mediolateral −2mm and dorsoventral in two depths −3,4 mm and −3,6 mm according to mouse brain atlas^39^. A total of 4,25 μg (4 μl) of human recombinant α-Synuclein preformed fibrils (PFF) of three different types: (a) wt-PFF, (b) P-PFF and (c) S129A-PFF were injected at a constant flow rate of 0.27 μl/min. Equal volume PBS was used for control animals. An interval of 5 min was maintained between the two dorsoventral depths and the needle was slowly removed 5 min after the injection procedure completed.

### Immunofluorescence analysis

For immunohistochemical analysis, animals were anesthetized by isoflurane surgical anesthesia and perfused intracardially with ice-cold PBS followed by 4% paraformaldehyde in PBS and post-fixed overnight. Brains were frozen in iso-pentane at −45 °C and stored at −80 ^o^C. Fluorescent immunohistochemistry was performed in free-floating sections of 35 μm. The sections were treated with antigen retrieval solution (citrate buffer, pH = 6) at 80 °C for 30 min. For the rodent specific D37A6, the human specific 211 α-Synuclein antibodies, and for double labeling with the SynO2 and α-Synuclein (phospho Ser 129) antibodies, sections were treated with Proteinase K (PK) (Sigma-Aldrich, USA) 5 μg/ml in PBS for 10 min at 25 ^o^C to expose antigenic sites^40^ To validate whether pathological α-Synuclein accumulations were PK resistant, sections were incubated with PK (5 μg/ml in PBS) at 37 °C (see Supplementary Fig. S3 and S6). For immunohistochemical staining with the anti-TH and anti-VMAT2 primary antibodies 3,3′-diaminobenzidine (DAB) (Dako, Denmark) was used as a chromogen, as previously described^41^. For immunocytochemistry cortical neurons were fixed in 4% (wt/vol) formaldehyde/4% (wt/vol) sucrose^42^. The antibodies used are shown in Table 1.

**Table 1:** List of primary antibodies used.

Protein	Company	Cat. No.	Working dilution
α-Synuclein C20 (human, mouse, rat)	Santa Cruz polyclonal	sc-7011	1:800 IHC
			1:1000 WB
α-Synuclein Syn1 (human, mouse, rat)	BD Transductions monoclonal	610787	1:500 WB
α-Synuclein Syn211 (human)	Abcam monoclonal	ab80627	1:20,000 IHC
			1:1000 WB
α-Synuclein D37A6 (mouse, rat)	Cell Signaling monoclonal	4179	1:500 IHC
			1:300 ICH
α-Synuclein (clone 5C2 human)	Acris monoclonal	Sm6028	1:100 IHC
α-Synuclein 4B12 (human)	GeneTex monoclonal	GTX21904	1:1000 WB
α-Synuclein LB509 (human)	BioLegend monoclonal	115–121	1:500 ICH
α-Synuclein (phospho S129)	ABCAM monoclonal	51253	1:2000 IHC
			1:1000 WB
Phosphorylated α-Synuclein, (Clone 64)	WAKO monoclonal	015–25191	1:1000 IHC
α-Synuclein (Syn O2)	Gift from Omar el Agnaf monoclonal	420 μg/ml	1:500 IHC
β-Actin	Cell Signaling monoclonal	4970	1:1000 WB
β Tubulin III	Sigma monoclonal	T8578	1:1000 ICH
GAPDH	Millipore monoclonal	MAB 374	1:5000 WB
P62	MBL polyclonal	PM045	1:1000 IHC
Snk (H90)	Santa Cruz polyclonal	sc-25421	1:500 WB
γ-tubulin	Sigma monoclonal	T5326	1:1000 WB
Tyrosine hydroxylase (TH)	Millipore monoclonal	MAB 318	1:2000 IHC
Tyrosine hydroxylase (TH)	Millipore polyclonal	MAB152	1:2000 IHC
Ubiquitin polyclonal	DAKO	Z0458	1:1000 IHC
VMAT2	ImmunoStaR polyclonal	20042	1:1500 IHC

### Confocal Microscopy

Fluorescent images were obtained with a Leica SP5-II upright confocal microscope and processed using the LAS AF (Leica Microsystems) and the Fiji v2.0.0 software. A protocol with sequential image acquisition was used. For mapping pathological accumulation within SNpc three coronal sections from each animal, corresponding to −2,82 mm, −3,1 mm and −3,38 mm anteroposterior distance relative to bregma, were selected. For measuring pathological accumulations in the cortex 6 sections/animal of 35 μm thickness were collected every 6, between 1,1 mm–0,1 mm anteroposterior distance relative to bregma. For the counting of pathological accumulations within nigral neurons and measuring the fluorescence intensity in the cortex the Fiji v 2.0.0 software was used. The fluorescence intensity of the endogenous α-Synuclein in the primary cultures was measured from a single 2D image which was created by projecting the confocal image stack using the Fiji z-projection function with the “sum slices” option^43^. The video file was created using the Volocity 3D image analysis software (Perkin Elmer, Waltham, MA).

### Stereology

The total number of TH-positive neurons in the SNpc was counted using a designed-base stereology, which uses Optical Fractionator as a method to perform quantification. The counting frame and systematic random sampling were applied for unbiased counting. For stereological analysis a total number of 5–6 animals per group was used. For each animal 6–8 sections of 35 μm thickness were collected every four throughout the rostro-caudal axis of the SN and counted. The counting contour was outlined with a 2.5x objective and counting was performed using a 63x glycerol immersion objective. Stereo Investigator v10.0 software (MBF Bioscience, USA) was used for the counting and the analysis with the following settings: Optical dissector Height (10 μm), grid size (80–100 μm) and counting frame (50 μm). A coefficient of error (Gundersen, m = 1) of ≤0.1 was accepted.

### Biochemical analysis

A two-step protein extraction protocol was followed. First the tissue was dissolved (7 ml/gr) in 1%Triton-X extraction buffer (150 mM NaCl, 50 mM Tris pH 7.6, 1% TritonX-100), sonicated and centrifuged at 100,000 *g* for 30 min. The supernatant (Triton-X-soluble fraction) was carefully removed. The pellet was washed twice with the Triton-X buffer and dissolved in 1% SDS RIPA buffer (50 mMTris, pH 8.0, 150 mMNaCl, 5 mM EDTA, 1%NP-40, 0.5% sodium deoxycholate, and 1% SDS). The supernatant recovered represents the SDS soluble fraction. Protease and phosphatase inhibitors (Roche, Switzerland) were added. Protein concentration was estimated by the Bradford and DC protein assay according to the manufacturer’s instructions (Bio-Rad, USA). The primary antibodies used are shown in Table 1. For the densitometry of immunoreactive bands the Gel Analyzer v1.0 software was used.

### High-performance liquid chromatography

Following cervical dislocation, DA was measured in the brain tissue homogenates with reverse phase ion-pair chromatography on an isocratic pump (YL9112) coupled with an electrochemical detector (BASi LC-EC) as previously described^44^. Briefly, the column used was YMC Triart C18 100 × 2 mm, 3 μm particle size, HPLC software (Clarity, UK) was used to quantify levels.

### IL-10, IFN-γ, IL-6 and TNF-α measurement in mouse striatum homogenates

Following anesthesia of animals by overdose administration of a ketamine (Merial)/xylazine (Bayer) cocktail by intraperitoneal injection, the brain was removed and mouse striatum was isolated and snap-frozen on dry ice. Samples were placed in a concentration of 50 mg of tissue per 1 ml of sterile HBSS containing protease inhibitors (Sigma-Aldrich, USA) and homogenized using a T-8 homogenizer (IKA-WERKE). The homogenates were centrifuged at 400 g for 15 minutes at 4 °C and the supernatants were collected and stored at −80 °C. IL-10, IFN-γ, IL-6 and TNF-α levels in the mouse striatum homogenates were measured using an IL-10, IFN-γ, IL-6 and TNF-a commercially available ELISA Kit (R&D Systems, USA) according to the manufacturer’s instructions. To verify specificity of effects in our study, two batches of PFF were used.

### Flow-cytometry analysis

Following anesthesia of animals by overdose administration of a ketamine (Merial)/xylazine (Bayer) cocktail by intraperitoneal injection, the ipsilateral mouse striatum was dissected, passed through 70 μm nylon cell strainers (BD Falcon) and single-cell suspensions were generated. 2 × 10^5^ total cells were stained with fluorescently-labeled antibodies against mouse CD45-FITC (Clone:30-F11) (1:100), CD45-PE (Clone:30-F11) (1:100), CD45-PE-Cyanine 5 (Clone:30-F11) (1:100), CD11b-PE-Cyanine 5 (Clone:M1/70) (1:100), CD11b-PE-Cyanine 7 (Clone:M1/70) (1:100), Arginase-1 (Arg-1)-FITC (R&D Systems) (1:100), nitric oxide synthase 2 (NOS-2)-PE Cyanine 7 (Clone: CXNFT) (1:100), MHCII- I-Ab-FITC (Clone:AF6–120.1) (1:100), CD86-PE (Clone:GL1) (1:100) (eBioscience) for 30 minutes. For intracellular IL-10 staining, 10^6^ cells were stained with fluorescently-labeled antibody against mouse IL-10-PE (Clone: JES5–16E3) (1:100), according to the manufacturer’s instructions (BD Biosciences). FACS acquisition was performed with the cytometer Cytomics FC500 (Beckman Coulter) and the data were analyzed using the FlowJo software 8.7 (Tree Star, Inc., Ashland, OR).

### Primary cultures of cortical neurons

Primary cortical neurons were prepared from embryonic day E16-E17 mouse brains (see supplementary Materials and Methods). Cultures at 6 DIV were treated with all three PFF types (0,4 μg/10^5^ cells) for 8 h and 24 h. After mild trypsinization with 0,5% Trypsin-EDTA for 5 minutes to remove the excess of unbound material, cells were lysed in STET lysis buffer (50 mM Tris (pH 7.6), 150 mM NaCl, 1% Triton-X, 2 mM EDTA), centrifuged at 16,000 g for 40 min at 4 °C and the supernatant recovered (Triton-X soluble fraction). Protease and phosphatase inhibitors were added. The pellet was resuspended in 1% Sarkosyl, sonicated and re-centrifuged (Sarkosyl soluble fraction). To verify specificity of effects in our study, two batches of PFF were used. Protein concentration was estimated by the Bradford assay. To monitor the effects of the fibrilar types on the endogenous α-Synuclein, primary cortical neurons were treated with all three PFF types for 5 and 8 days. Fixed cultures were immunostained and subsequently analyzed.

### Behavioral analysis

Mice injected with either the three fibrilar types or PBS-control were tested in a comprehensive behavioral test battery to assess gross and fine motor deficits at 60 dpi, prior to sacrifice. The following tests were conducted in the same order for all subjects: open field, rotarod, challenging beam traversal, pole test, and grip strength (see Supplementary Materials and Methods).

### Statistical analysis

For data analysis GraphPad Prism 5 software was used. Statistics were performed using the One-way ANOVA test followed by Tukey’s Multiple Comparison test, and two-tailed paired Student’s t-test. For data presented in Fig. 8c and d and Supplementary Fig. S13c, non-parametric Kruskal-Wallis test was applied followed by Dunn’s multiple comparison test due to lack of normality of distributions (Continuous data were checked for normality of distribution and homogeneity of variances by Shapiro Wilk’s and Levene’s tests respectively). Values were considered significant different for *P* < 0.05. *P < 0.05, **P < 0.01 and ***P < 0.001.

### Data availability

All data generated or analyzed during this study are included in this published article (and its Supplementary Information files).

### Ethical approval

The study was performed in the Laboratory Animal Unit of Centre of the Biomedical Research Foundation of the Academy of Athens. The competent Regional Veterinary authority approved the experimental protocol in accordance to Greek legislation (Presidential Decree 56/2013, in compliance with the European Directive 2010/63).

## Electronic supplementary material

Supplementary information

Supplementary video file

## References

[1] Polymeropoulos MH (1997). Mutation in the alpha-synuclein gene identified in families with Parkinson’s disease. Science.

[2] Singleton AB (2003). alpha-Synuclein locus triplication causes Parkinson’s disease. Science.

[3] Fujiwara H (2002). alpha-Synuclein is phosphorylated in synucleinopathy lesions. Nature cell biology.

[4] Kahle PJ (2002). Hyperphosphorylation and insolubility of alpha-synuclein in transgenic mouse oligodendrocytes. EMBO reports.

[5] Anderson JP (2006). Phosphorylation of Ser-129 is the dominant pathological modification of alpha-synuclein in familial and sporadic Lewy body disease. The Journal of biological chemistry.

[6] Tenreiro S, Eckermann K, Outeiro TF (2014). Protein phosphorylation in neurodegeneration: friend or foe?. Frontiers in molecular neuroscience.

[7] Smith WW (2005). Alpha-synuclein phosphorylation enhances eosinophilic cytoplasmic inclusion formation in SH-SY5Y cells. The Journal of neuroscience: the official journal of the Society for Neuroscience.

[8] Paleologou KE (2008). Phosphorylation at Ser-129 but not the phosphomimics S129E/D inhibits the fibrillation of alpha-synuclein. The Journal of biological chemistry.

[9] Fiske M (2011). Contribution of Alanine-76 and Serine Phosphorylation in alpha-Synuclein Membrane Association and Aggregation in Yeasts. Parkinson’s disease.

[10] Kragh CL (2009). Alpha-synuclein aggregation and Ser-129 phosphorylation-dependent cell death in oligodendroglial cells. The Journal of biological chemistry.

[11] Kuwahara T, Tonegawa R, Ito G, Mitani S, Iwatsubo T (2012). Phosphorylation of alpha-synuclein protein at Ser-129 reduces neuronal dysfunction by lowering its membrane binding property in Caenorhabditis elegans. The Journal of biological chemistry.

[12] Febbraro F (2013). Ser129D mutant alpha-synuclein induces earlier motor dysfunction while S129A results in distinctive pathology in a rat model of Parkinson’s disease. Neurobiology of disease.

[13] Peelaerts W (2015). alpha-Synuclein strains cause distinct synucleinopathies after local and systemic administration. Nature.

[14] Luk KC (2012). Pathological alpha-synuclein transmission initiates Parkinson-like neurodegeneration in nontransgenic mice. Science.

[15] Luk KC (2012). Intracerebral inoculation of pathological alpha-synuclein initiates a rapidly progressive neurodegenerative alpha-synucleinopathy in mice. The Journal of experimental medicine.

[16] Vaikath NN (2015). Generation and characterization of novel conformation-specific monoclonal antibodies for alpha-synuclein pathology. Neurobiology of disease.

[17] Pan WX, Mao T, Dudman JT (2010). Inputs to the dorsal striatum of the mouse reflect the parallel circuit architecture of the forebrain. Frontiers in neuroanatomy.

[18] Olson KE, Gendelman HE (2016). Immunomodulation as a neuroprotective and therapeutic strategy for Parkinson’s disease. Current opinion in pharmacology.

[19] Bennett ML (2016). New tools for studying microglia in the mouse and human CNS. Proceedings of the National Academy of Sciences of the United States of America.

[20] Mougenot AL (2012). Prion-like acceleration of a synucleinopathy in a transgenic mouse model. Neurobiology of aging.

[21] Masuda-Suzukake M (2013). Prion-like spreading of pathological alpha-synuclein in brain. Brain: a journal of neurology.

[22] Recasens A (2014). Lewy body extracts from Parkinson disease brains trigger alpha-synuclein pathology and neurodegeneration in mice and monkeys. Annals of neurology.

[23] Bjorklund A, Dunnett SB (2007). Dopamine neuron systems in the brain: an update. Trends in neurosciences.

[24] Brundin P, Melki R, Kopito R (2010). Prion-like transmission of protein aggregates in neurodegenerative diseases. Nature reviews. Molecular cell biology.

[25] Visanji NP, Brooks PL, Hazrati LN, Lang AE (2013). The prion hypothesis in Parkinson’s disease: Braak to the future. Acta neuropathologica communications.

[26] Guo JL, Lee VM (2014). Cell-to-cell transmission of pathogenic proteins in neurodegenerative diseases. Nature medicine.

[27] Osterberg VR (2015). Progressive aggregation of alpha-synuclein and selective degeneration of lewy inclusion-bearing neurons in a mouse model of parkinsonism. Cell reports.

[28] Sacino, A. N. *et al*. Proteolysis of alpha-Synuclein Fibrils in the Lysosomal Pathway Limits Induction of Inclusion Pathology. *Journal of neurochemistry*10.1111/jnc.13743 (2016). 10.1111/jnc.13743 PMC5452425 27424880

[29] Samuel F (2016). Effects of Serine 129 Phosphorylation on alpha-Synuclein Aggregation, Membrane Association, and Internalization. The Journal of biological chemistry.

[30] Schreurs S (2014). *In vitro* phosphorylation does not influence the aggregation kinetics of WT alpha-synuclein in contrast to its phosphorylation mutants. International journal of molecular sciences.

[31] Ma MR, Hu ZW, Zhao YF, Chen YX, Li YM (2016). Phosphorylation induces distinct alpha-synuclein strain formation. Scientific reports.

[32] Mao, X. *et al*. Pathological alpha-synuclein transmission initiated by binding lymphocyte-activation gene 3. *Science* **353**, 10.1126/science.aah3374 (2016). 10.1126/science.aah3374 PMC5510615 27708076

[33] Lee KW (2011). Enhanced phosphatase activity attenuates alpha-synucleinopathy in a mouse model. The Journal of neuroscience: the official journal of the Society for Neuroscience.

[34] Bossu P, Spalletta G, Caltagirone C, Ciaramella A (2015). Myeloid Dendritic Cells are Potential Players in HumanNeurodegenerative Diseases. Frontiers in immunology.

[35] Christiansen JR, Olesen MN, Otzen DE, Romero-Ramos M, Sanchez-Guajardo V (2016). alpha-Synuclein vaccination modulates regulatory T cell activation and microglia in the absence of brain pathology. Journal of neuroinflammation.

[36] Ardah MT (2015). Ginsenoside Rb1 inhibits fibrillation and toxicity of alpha-synuclein and disaggregates preformed fibrils. Neurobiology of disease.

[37] Ardah MT (2014). Structure activity relationship of phenolic acid inhibitors of alpha-synuclein fibril formation and toxicity. Frontiers in aging neuroscience.

[38] Mbefo MK (2010). Phosphorylation of synucleins by members of the Polo-like kinase family. The Journal of biological chemistry.

[39] Paxinos, G. & Franklin, K. B. The Mouse Brain in Stereotaxic Coordinates, 2nd Edn. *Elsevier Academic Press San Diego* (2004).

[40] Mamais A (2013). Divergent alpha-synuclein solubility and aggregation properties in G2019S LRRK2 Parkinson’s disease brains with Lewy Body pathology compared to idiopathic cases. Neurobiology of disease.

[41] Sotiriou E, Vassilatis DK, Vila M, Stefanis L (2010). Selective noradrenergic vulnerability in alpha-synuclein transgenic mice. Neurobiology of aging.

[42] Volpicelli-Daley LA, Luk KC, Lee VM (2014). Addition of exogenous alpha-synuclein preformed fibrils to primary neuronal cultures to seed recruitment of endogenous alpha-synuclein to Lewy body and Lewy neurite-like aggregates. Nature protocols.

[43] Schindelin J (2012). Fiji: an open-source platform for biological-image analysis. Nature methods.

[44] Xilouri M (2013). Boosting chaperone-mediated autophagy *in vivo* mitigates alpha-synuclein-induced neurodegeneration. Brain: a journal of neurology.

