# Supplementary material for: Phosphorylated exogenous alpha-synuclein fibrils exacerbate pathology and induce neuronal dysfunction in mice
Source: Sci Rep. 2017 Nov 28;7:16533. doi: 10.1038/s41598-017-15813-8 (PMC5705684; doi:10.1038/s41598-017-15813-8)
Supplement: Supplementary file 1 — Supplementary information [file 41598_2017_15813_MOESM1_ESM.pdf]

SUPPLEMENTARY INFORMATION

Phosphorylated exogenous alpha-synuclein fibrils exacerbate pathology and induce neuronal dysfunction in mice

Karampetsou Mantia, Ardah Mustafa T, Semitekolou Maria, Polissidis Alexia, Samiotaki Martina, Kalomoiri Maria, Majbour Nour, Xanthou Georgina, El-Agnaf M.A. Omar and Vekrellis Kostas

Supplementary Table

Sequence	# PSMs	Modifications	phosphoRS Site Probabilities	XCorr	Charge	MH+ [Da]	ΔM [ppm]
DMPVDPDNEAYEmPsEEGYQ	2	M13(Oxidation); S15(Phospho)	Y(11): 0.0; S(15): 98.8; Y(19): 1.2	3.77	2	2411.85967	1.17
DMPVDPDNEAYEmPSEEGYQ	1	M13(Oxidation)		3.55	3	2331.89463	1.77
DMPVDPDNEAYEMPsEEGYQ	6	S15(Phospho)	Y(11): 0.0; S(15): 99.0; Y(19): 1.0	3.77	3	2395.86655	1.93
DNEAYEmPsEEGYQ	2	M7(Oxidation); S9(Phospho)	Y(5): 0.0; S(9): 99.2; Y(13): 0.8	2.10	2	1757.59184	1.92
DQLGKnEEGAPQEGILE	4	N6(Deamidated)		2.63	3	1827.85855	-1.22
DQLGKNEEGAPQEGILE	24			3.86	2	1826.87920	1.33

Supplementary Table Human α-Synuclein AspN-specific peptides identified and characterized from digested P-PFF analyzed by LC-MS/MS.

Supplementary Figures

Supplementary Figure S1

a

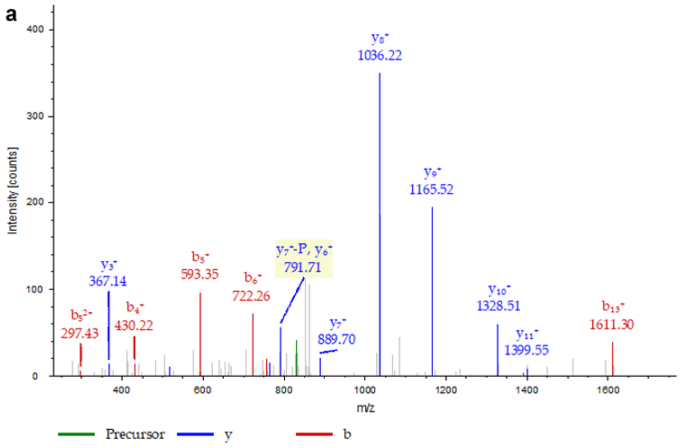

#1	b+	b2+	Seq.	y+	y2+	#2
1	116.03423	58.52075	D			14
2	230.07716	115.54222	N	1642.56153	821.78440	13
3	359.11976	180.06352	E	1528.51860	764.76294	12
4	430.15688	215.58208	A	1399.47600	700.24164	11
5	593.22020	297.11374	Y	1328.43888	664.72308	10
6	722.26280	361.63504	E	1165.37556	583.19142	9
7	869.29821	435.15274	M-Oxidation	1036.33296	518.67012	8
8	966.35098	483.67913	P	889.29754	445.15241	7
9	1133.34934	567.17831	S-Phospho	792.24477	396.62602	6
10	1262.39194	631.69961	E	625.24641	313.12684	5
11	1391.43454	696.22091	E	496.20381	248.60554	4
12	1448.45601	724.73164	G	367.16121	184.08424	3
13	1611.51933	806.26330	Y	310.13974	155.57351	2
14			Q	147.07642	74.04185	1

DNEAYEMPSEEGYQ: M7-Oxidation (15.99492 Da), S9-Phospho (79.96633 Da)

b

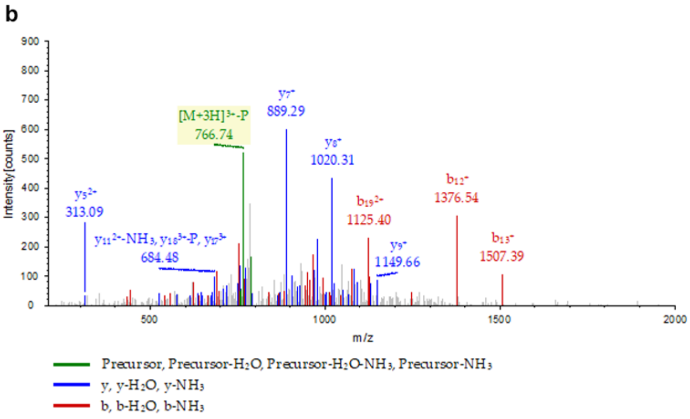

#1	b+	b2+	b3+	Seq.	y+	y2+	y3+	#2
1	116.03423	58.52075	39.34959	D				20
2	247.07473	124.04100	83.02976	M	2280.83497	1140.92112	760.94984	19
3	344.12750	172.56739	115.38068	P	2149.79447	1075.40087	717.26968	18
4	443.19592	222.10160	148.40349	V	2052.74170	1026.87449	684.91875	17
5	558.22287	279.61507	186.74581	D	1953.67328	977.34028	651.89595	16
6	655.27564	328.14146	219.09673	P	1838.64633	919.82680	613.55363	15
7	770.30259	385.65493	257.43905	D	1741.59356	871.30042	581.20271	14
8	884.34552	442.67640	295.45336	N	1626.56661	813.78694	542.86039	13
9	1013.38812	507.19770	338.46756	E	1512.52368	756.76548	504.84608	12
10	1084.42524	542.71626	362.14660	A	1383.48108	692.24418	461.83188	11
11	1247.48856	624.24792	416.50104	Y	1312.44396	656.72562	438.15284	10
12	1376.53116	688.76922	459.51524	E	1149.38064	575.19396	383.79840	9
13	1507.57166	754.28947	503.19540	M	1020.33804	510.67266	340.78420	8
14	1604.62443	802.81585	535.54633	P	889.29754	445.15241	297.10403	7
15	1771.62279	886.31503	591.21245	S-Phospho	792.24477	396.62602	264.75311	6
16	1900.66539	950.83633	634.22665	E	625.24641	313.12684	209.08699	5
17	2029.70799	1015.35763	677.24085	E	496.20381	248.60554	166.07279	4
18	2086.72946	1043.86837	696.24800	G	367.16121	184.08424	123.05859	3
19	2249.79278	1125.40003	750.60244	Y	310.13974	155.57351	104.05143	2
20				Q	147.07642	74.04185	49.69699	1

DMPVDPDNEAYEMPSEEGYQ: S15-Phospho (79.96633 Da)

c

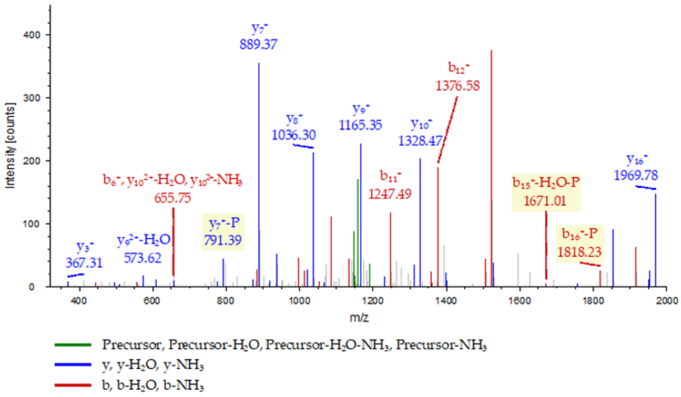

#1	b^+	b^{2+}	Seq.	y^+	y^{2+}	#2
1	116.03423	58.52075	D			20
2	247.07473	124.04100	M	2296.82989	1148.91858	19
3	344.12750	172.56739	P	2165.78939	1083.39833	18
4	443.19592	222.10160	V	2068.73662	1034.87195	17
5	558.22287	279.61507	D	1969.66820	985.33774	16
6	655.27564	328.14146	P	1854.64125	927.82426	15
7	770.30259	385.65493	D	1757.58848	879.29788	14
8	884.34552	442.67640	N	1642.56153	821.78440	13
9	1013.38812	507.19770	E	1528.51860	764.76294	12
10	1084.42524	542.71626	A	1399.47600	700.24164	11
11	1247.48856	624.24792	Y	1328.43888	664.72308	10
12	1376.53116	688.76922	E	1165.37556	583.19142	9
13	1523.56657	762.28692	M-Oxidation	1036.33296	518.67012	8
14	1620.61934	810.81331	P	889.29754	445.15241	7
15	1787.61770	894.31249	S-Phospho	792.24477	396.62602	6
16	1916.66030	958.83379	E	625.24641	313.12684	5
17	2045.70290	1023.35509	E	496.20381	248.60554	4
18	2102.72437	1051.86582	G	367.16121	184.08424	3
19	2265.78769	1133.39748	Y	310.13974	155.57351	2
20			Q	147.07642	74.04185	1

DMPVDPDNEAYEMPSEEGYQ: M13-Oxidation (15.99492 Da), S15-Phospho (79.96633 Da)

Supplementary Figure S1. Fragmentation spectrum and the annotated lists of b and y ions (fragments) of doubly-charged peaks corresponding to α-Synuclein peptides. **a** DNEAYEMPSEEGYQ modified with one methionine oxidation (15.99492 Da) and one phospho-group (79.96633 Da). **b** DMPVDPDNEAYEMPSEEGYQ modified with one phospho-group (79.96633 Da). **c** DMPVDPDNEAYEMPSEEGYQ modified with one methionine oxidation (15.99492 Da) and one phospho-group (79.96633 Da). b and y ion are shown in red and blue respectively while prominent neutral loss ions are shown in green.

Supplementary Figure S2

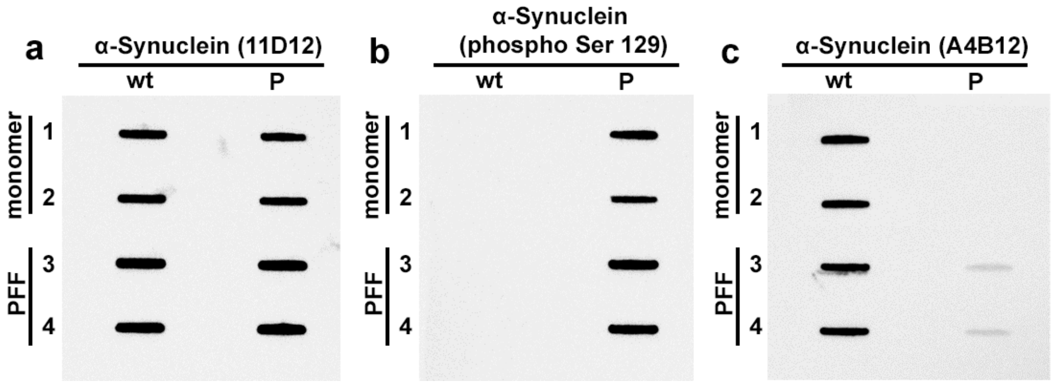

Supplementary Figure S2. Sonicated P-PFF are adequately phosphorylated by PLK2 kinase. 50 ng of sonicated wt-PFF and P-PFF as well as wt- and P- monomeric recombinant human α-Synuclein were loaded on a slot blot in duplicate. For the detection of different α-Synuclein species the following antibodies were used: **a** 11D12 (total, human specific[1]). **b** phospho Ser 129 (phosphorylated) and **c** A4B12 (non-phosphorylated).

Supplementary Figure S3

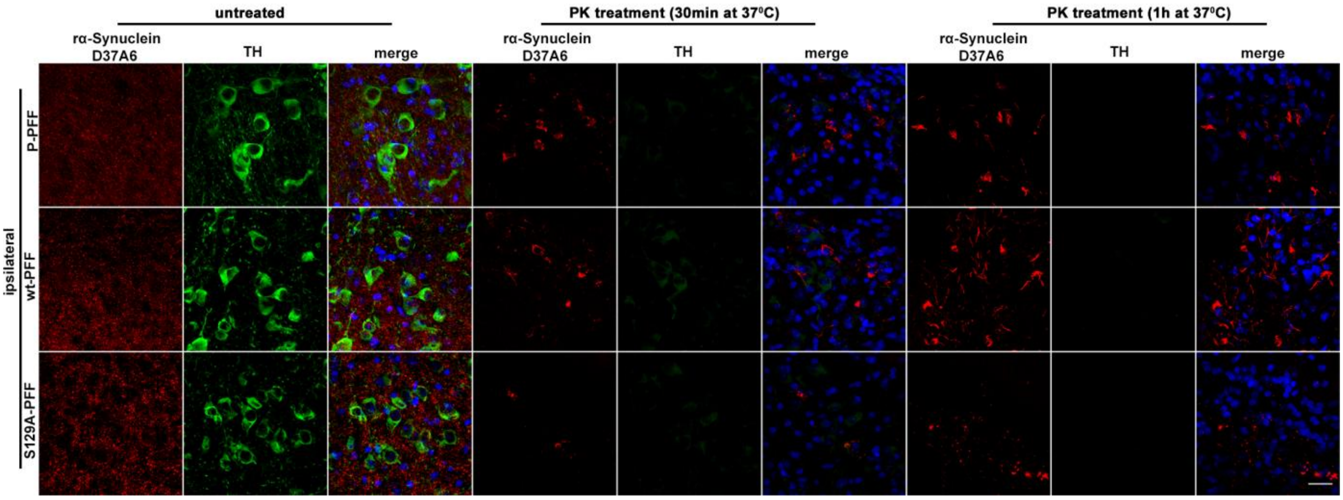

Supplementary Figure S3. Time course of treatment with PK in nigral sections of wt animals injected with the three fibrilar types P-, wt- and S129A-PFF. Pathological accumulations stained positive with the rodent specific α-Synuclein antibody (D37A6) following PK treatment at 37^0C for 30min and 1h. Note that under such treatment TH signal is lost. These PK-resistant accumulations are not revealed in untreated sections possibly because the antigen binding sites are not exposed for immunolabelling with the D37A6 antibody. Scale bar represents 25μm

Supplementary Figure S4

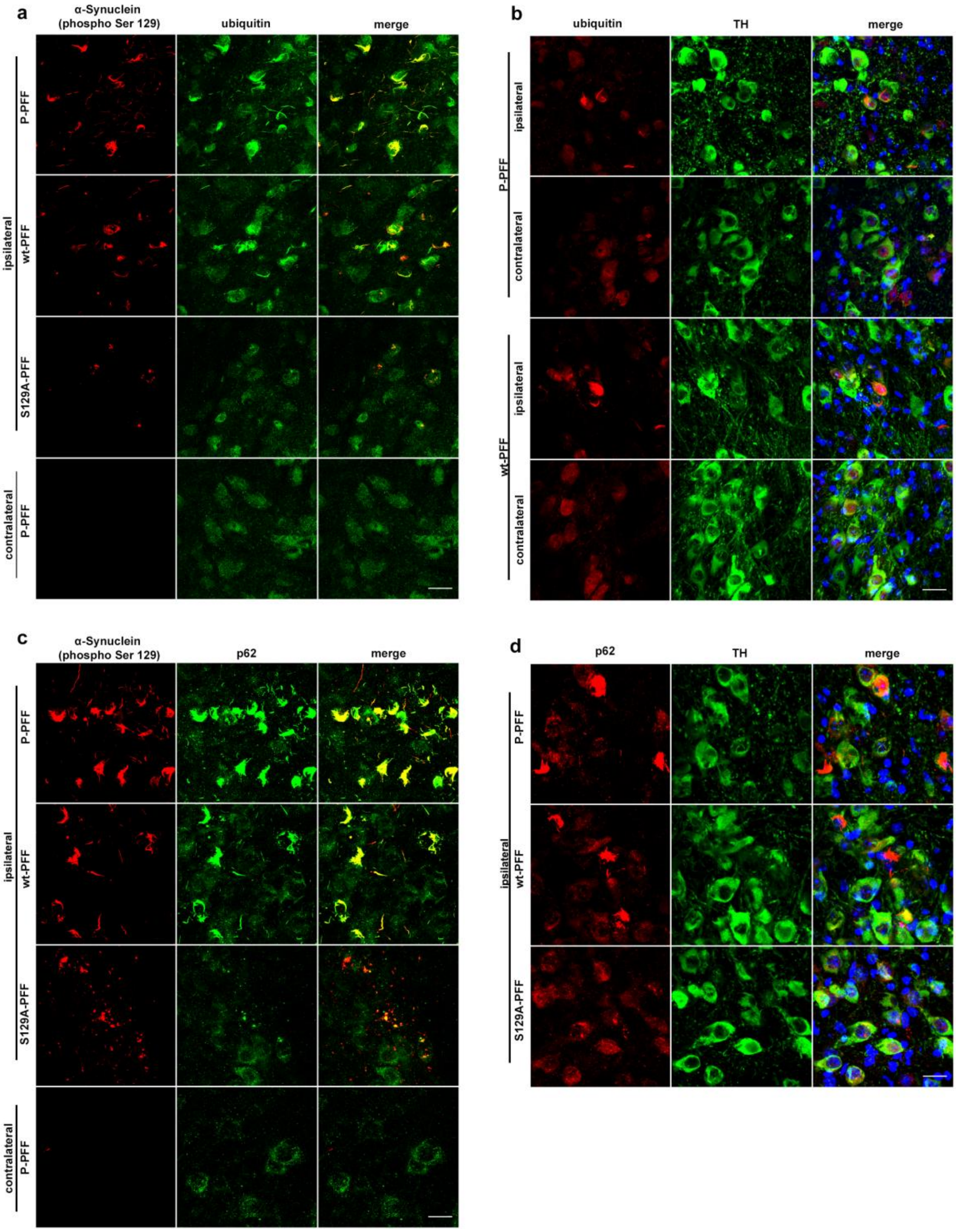

Supplementary Figure S4. Pathological accumulations share common features with LBs. a Phosphorylated inclusions present in nigral sections of P- and wt-PFF injected animals are also positively stained for ubiquitin. On the contrary, in S129A-

PFF injected animals double immunostaining against P-α-Synuclein and ubiquitin shows only partial co-localization. Contralateral side shows basal levels of ubiquitin expression (representative image from the contralateral SNpc of P-PPF injected animals).**b** To verify the overlapping staining pattern presented in (a**)**, nigral sections from P- and wt-PFF injected animals double-stained for ubiquitin and TH. Ubiquitin positive accumulations are evident within TH neurons. **c** P-α-Synuclein positive inclusions showed strong co-localization with p62 in nigral sections of P- and wt-PFF injected animals. Inclusions in the S129A injected animals partly co-localized with p62. Diffuse and punctate cytoplasmic staining of p62 was observed in the contralateral side. **d** The specificity of the overlapping staining (α-Synuclein (phospho Ser129)/p62) was further estimated by p62/TH double staining. Scale bars represent 25μm

Supplementary Figure S5

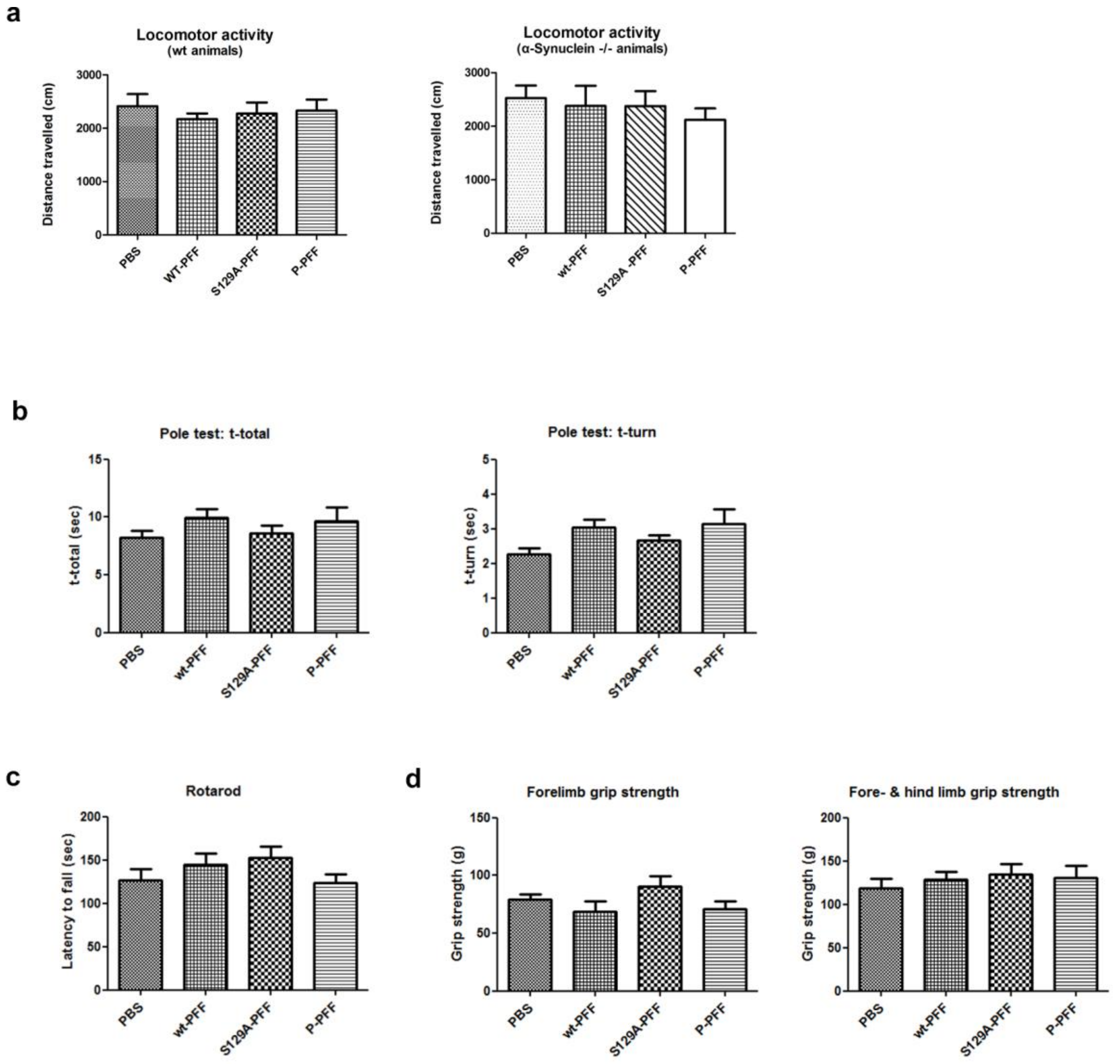

Supplementary Figure S5. 60 dpi behavioral assessments included a locomotor activity in wt and α-Synuclein null (-/-) animals**. b** pole test (average time to orient downward (t-turn, sec) and time to descend (t-total, sec), **c** rotarod (average latency to fall, sec) and **d** grip strength (average, g). None of the tests revealed significant differences (n=8-10 animals/group). Data represent mean values ±SEM. Differences were estimated using one-way ANOVA.

Supplementary Figure S6

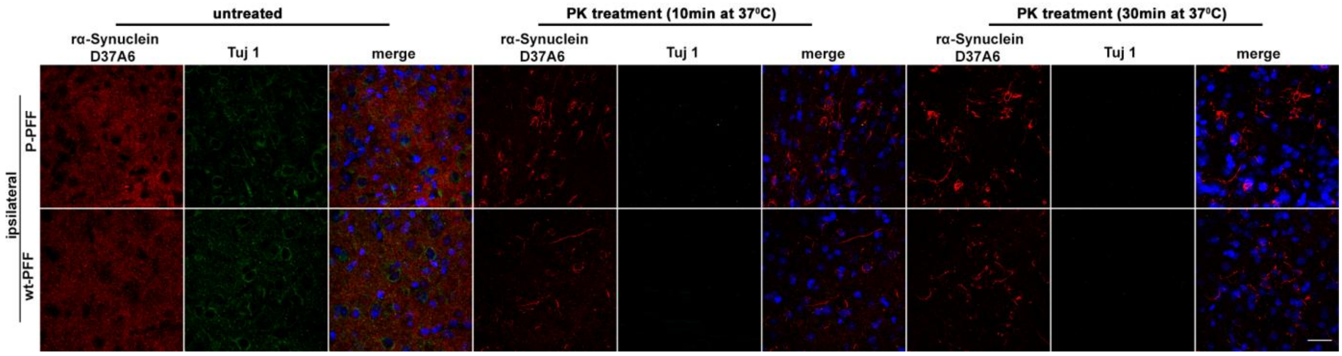

Supplementary Figure S6. Time course of treatment with PK in cortical sections of injected animals. PK resistant accumulations were stained positive for the rodent specific D37A6 α-Synuclein antibody in the ipsilateral cortex of P- and wt-PFF injected animals following PK treatment at 37^{0}C up to 30min. At this timepoint, there was no remaining Tuj 1 signal. Pathological D37A6 positive accumulations were undetectable in non-treated section. Scale bar represents 25µm.

Supplementary Figure S7

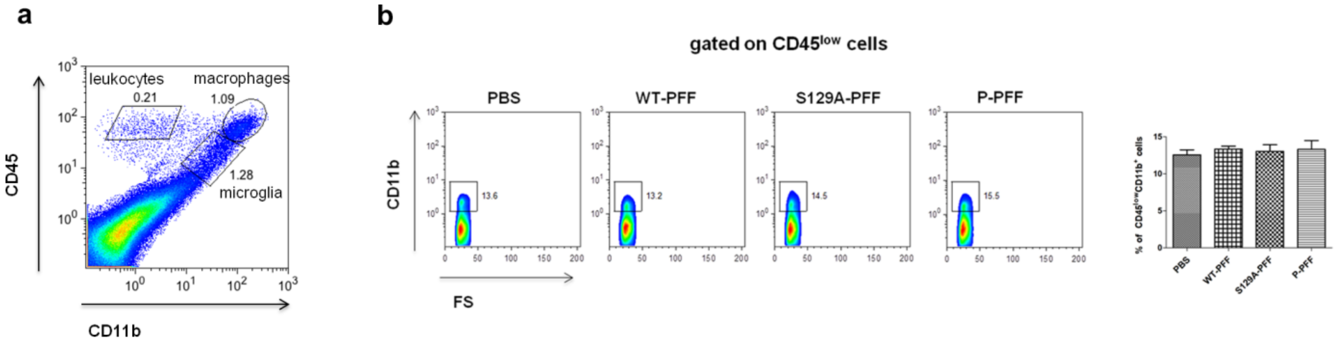

Supplementary Figure S7. a Gating strategy followed to distinguish microglia vs infiltrating peripheral macrophages based on the expression levels of CD45 and CD11b: microglia being CD11b^{+}CD45^{low} vs macrophages being CD11b^{+}CD45^{high}. **b** Cells obtained from ipsilateral striatal tissue of wt mice administered with PBS, wt-,

S129A- and P-PFF for 3 days, were stained and analyzed by flow-cytometry. Representative FACS plots showing CD11b^{+} expression gated on CD45^{low} cells, considered microglia[2]. Cumulative data showing the percentages of CD45^{low}CD11b^{+} cells are depicted on the right. Results are pooled from three independent experiments. Data represent mean values ±SEM. Differences were estimated using one-way ANOVA.

Supplementary Figure S8

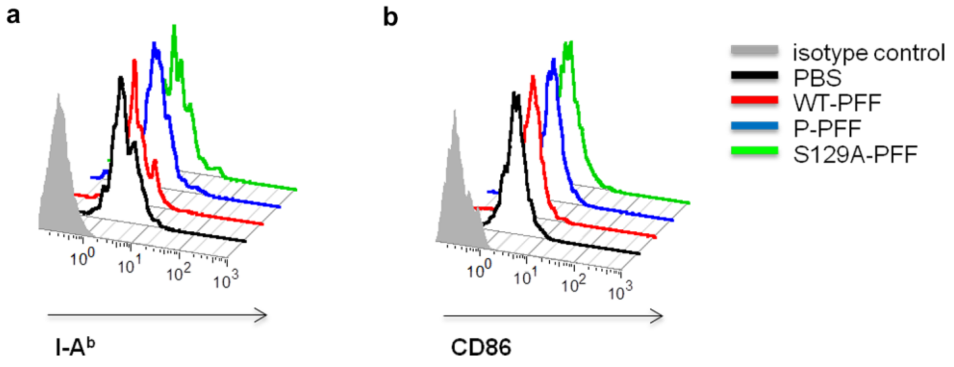

Supplementary Figure S8. Cells obtained from ipsilateral striatal tissue of wt mice administered with PBS, wt-, S129A- and P-PFF for 3 days, were stained and analyzed by flow-cytometry. FACS plots showing **a** I-Ab^{+} and **b** CD86^{+} expression gated on CD45^{high}CD11b^{+} macrophages. Shaded histograms represent isotype controls. Results are representative of two independent experiments.

Supplementary Figure S9

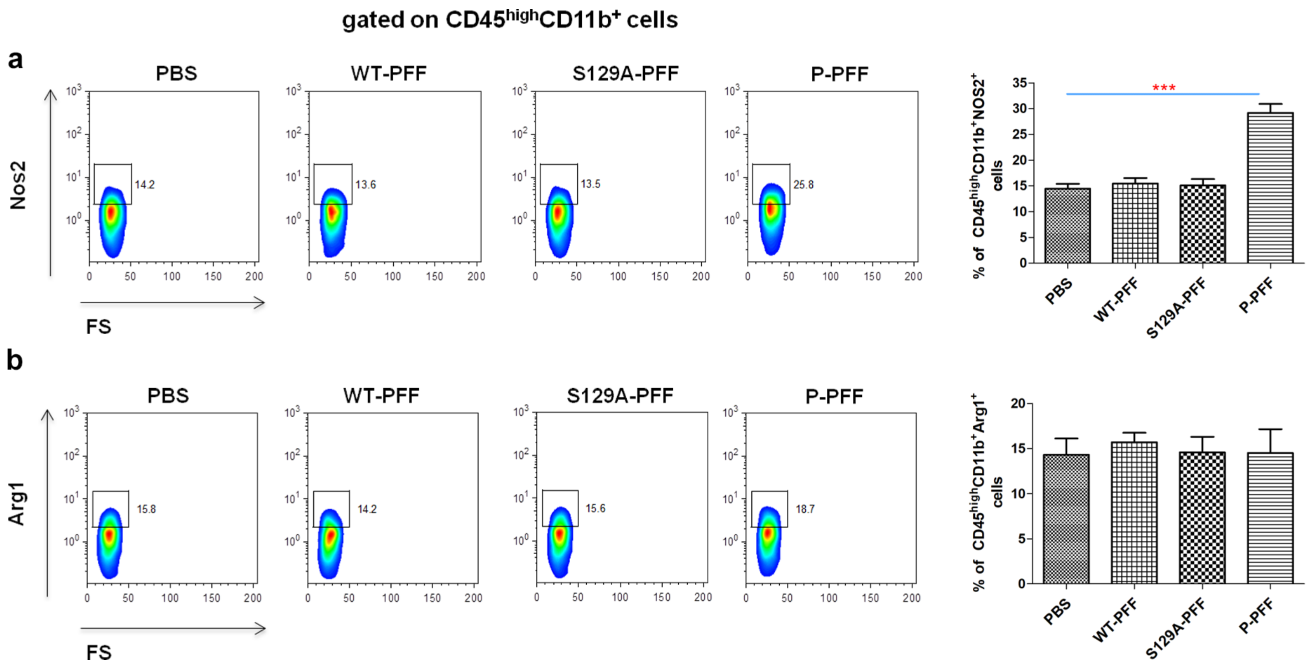

Supplementary Figure S9. Cells obtained from ipsilateral striatal tissue of wt mice administered with PBS, wt-, S129A- and P-PFF for 3 days, were stained and analyzed by flow-cytometry. Representative FACS plots showing **a** $Nos2^{+}$ and **b** $Arg1^{+}$ expression gated on $CD45^{high}CD11b^{+}$ macrophages. Cumulative data showing the percentages of $CD45^{high}CD11b^{+}Nos2^{+}$ and $CD45^{high}CD11b^{+}Arg1^{+}$cells are depicted on the right. Results are pooled from three independent experiments. Statistical analysis was performed by ANOVA followed by Tukey's post-hoc test. **a)**$p<0,0001$

Supplementary Figure S10

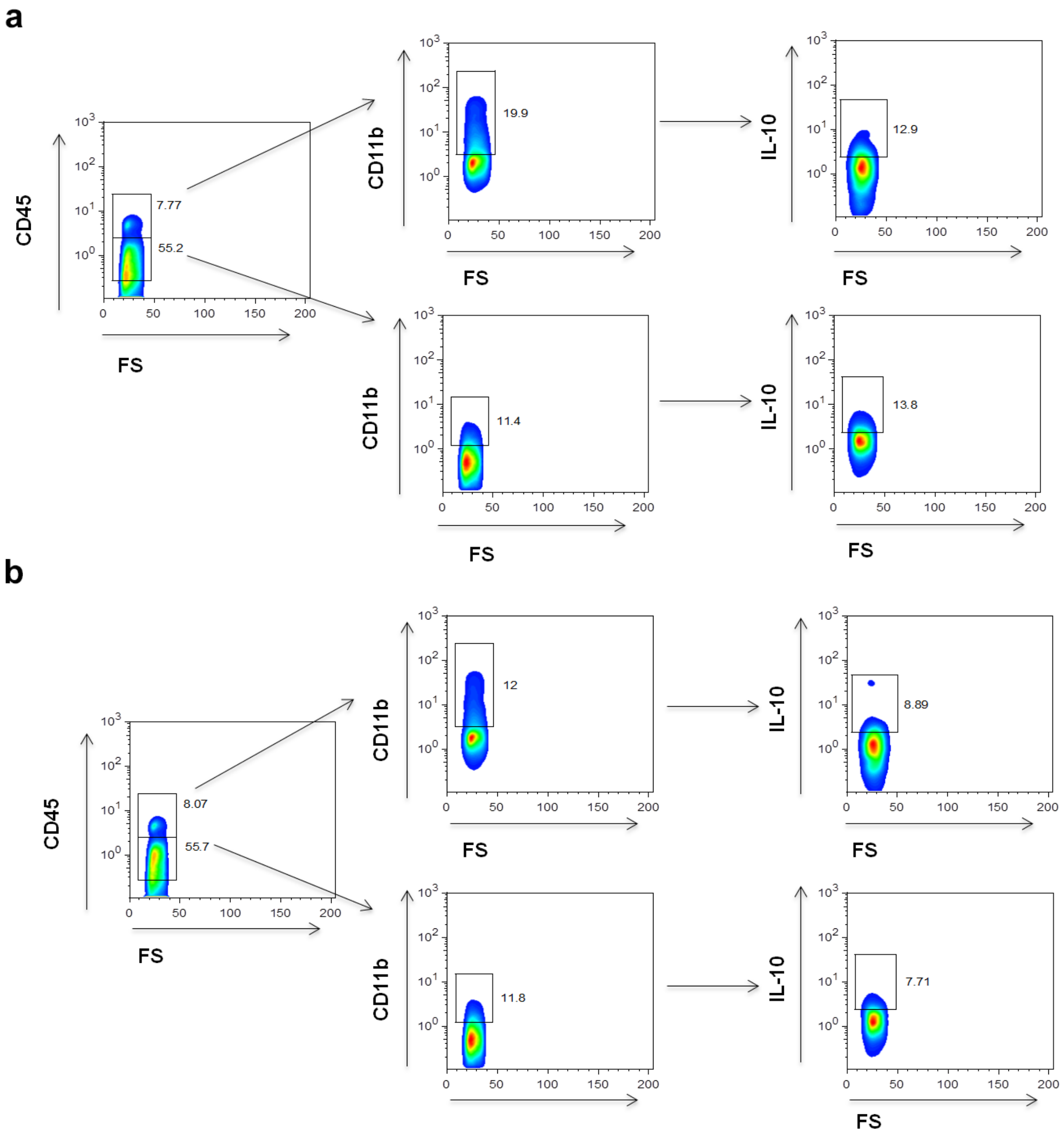

Supplementary Figure S10. Cells obtained from ipsilateral striatal tissue of wt mice administered with **a** wt-PFF or **b** P-PFF for 3 days, were stained and analyzed by flow-cytometry. FACS plots showing IL-10^{+} expression gated on $CD45^{high}CD11b^{+}$ or $CD45^{low}CD11b^{+}$ cells. Results are representative of two independent experiments.

Supplementary Figure S11

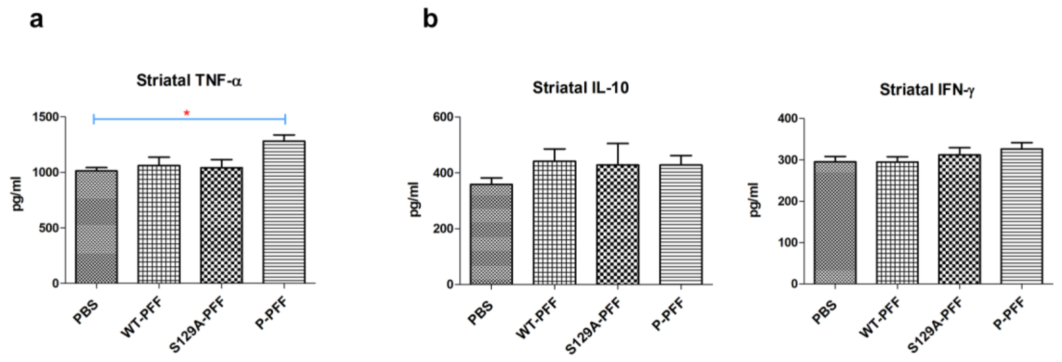

Supplementary Figure S11. a TNF-α **b** IL-10 and IFN-γ levels were measured in ipsilateral striatum homogenates by ELISA at 60 dpi. Data are expressed as mean ± SEM of triplicate wells. Data shown are pooled from four-six independent experiments. Statistical significance was obtained by ANOVA followed by Tukey's post-hoc test. **a)**p=0,0232

Supplementary Figure S12

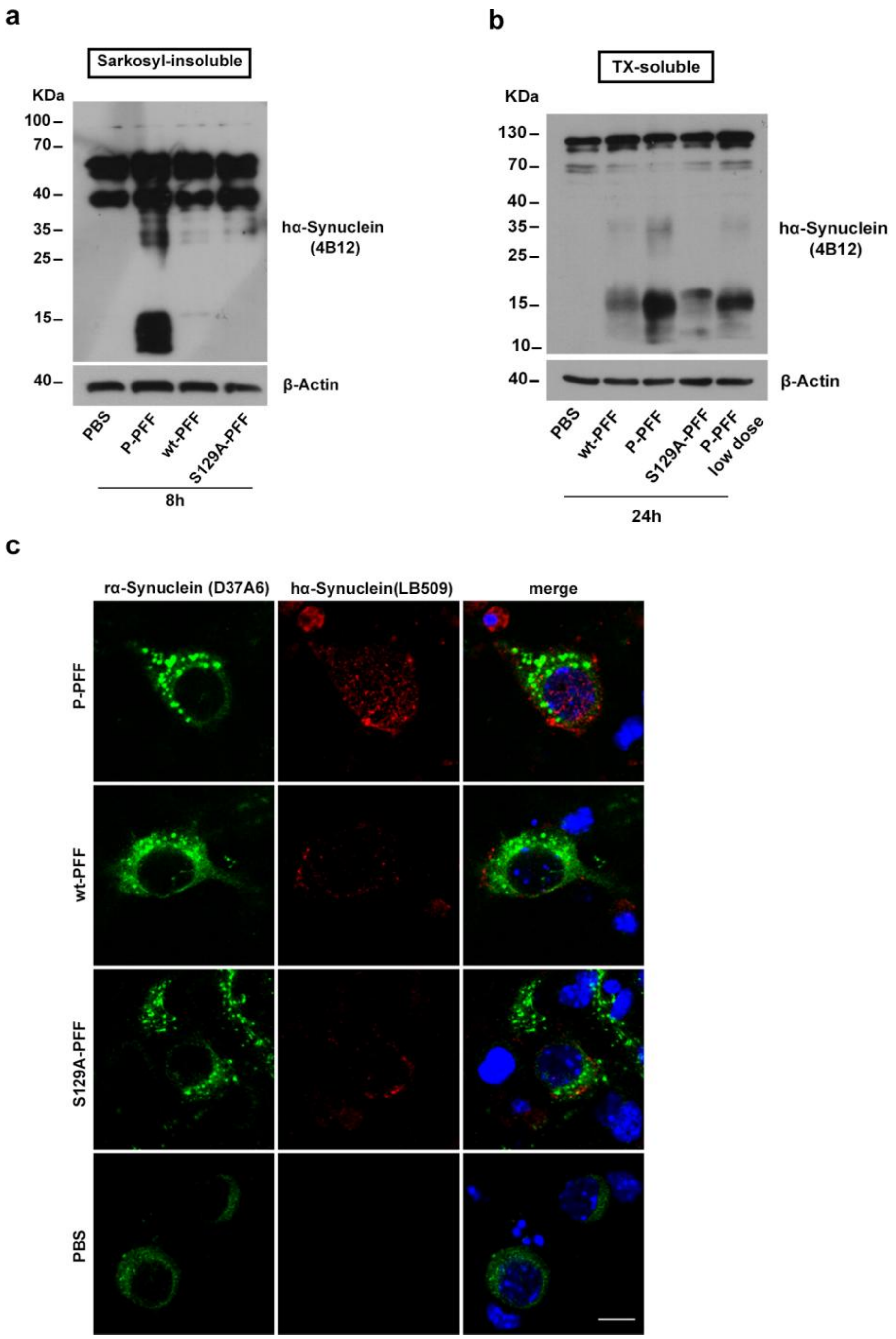

Supplementary Figure S12. a Mouse primary cortical cultures (6div) were treated with PFF for 8 hours. Following Triton-X and Sarkosyl extraction the insoluble pellet was immunoblotted with the human specific α-Synuclein antibody 4B12. P-PFF presence was more abundant compared to wt- and S129A-PFF. β-actin was used as a

loading control. **b** Mouse primary cortical cultures were treated with half the concentration (0,2μg/10^5 cells) of P-PFF compared to the other types for 24 hours. Following Triton-X extraction, internalized PFF were visualized with the human specific α-Synuclein antibody 4B12. Even at lower concentration P-PFF exhibited increased ability of uptake by neurons. β-actin was used as a loading control. **c** Seeding of endogenous α-Synuclein following treatment of mouse cortical cultures with PFF.6-day-old mouse cortical cultures were treated with P-, wt-, and S129A-PFF (0,4μg/10^5 cells) for 5 days. Seeding of the endogenous α-Synuclein, as depicted by foci formation, was visualized by immunocytochemistry using the rodent specific antibody (D37A6). Exogenously added human PFF were detected by co-staining with the human specific antibody LB509. TO-PRO-3 (blue) was used as a cell nuclear marker. Scale bar represents 10μm.

Supplementary Figure S13

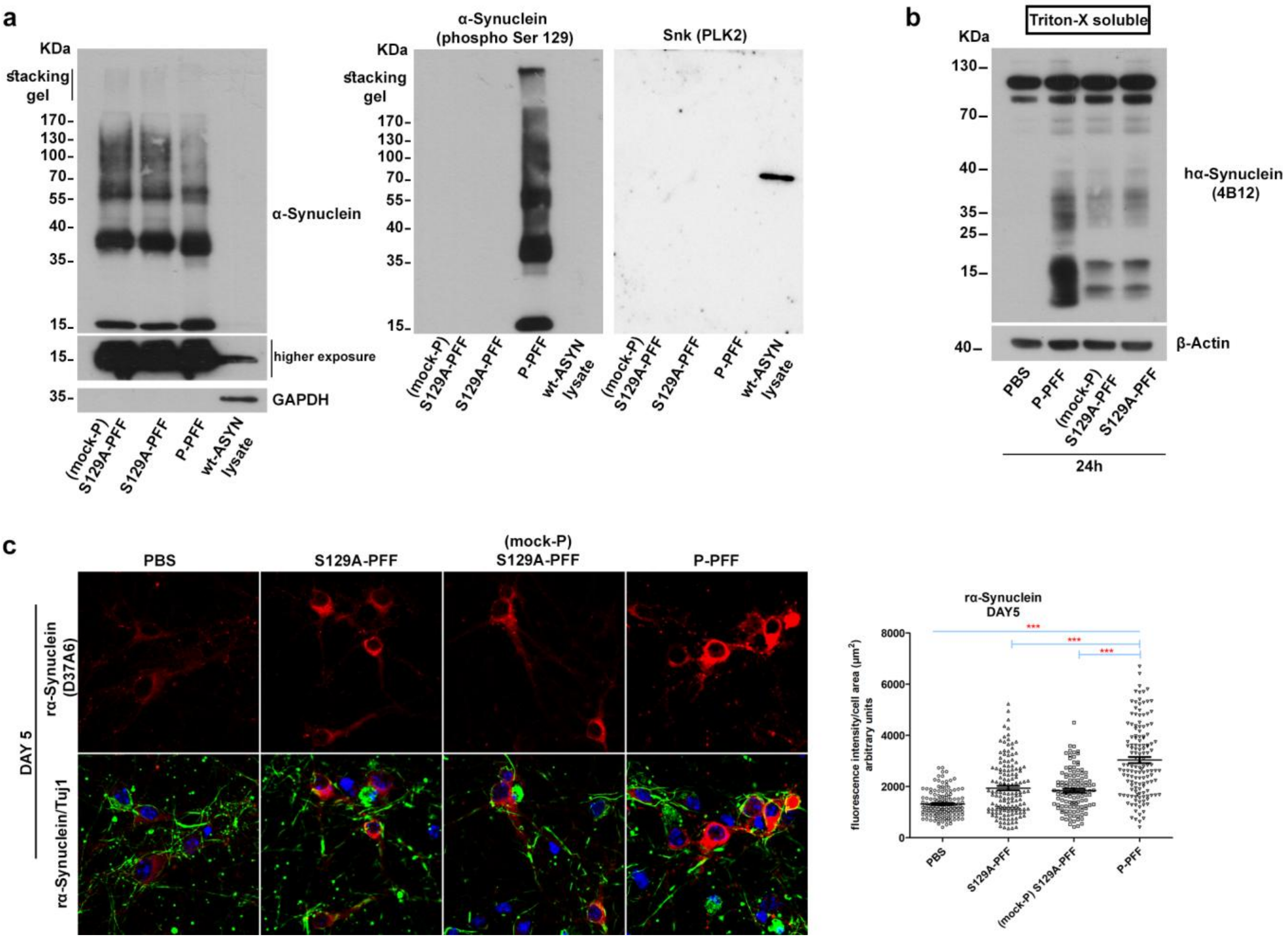

Supplementary Figure S13 a Western blotting analysis for P-, S129A- and (mock-P) S129A-PFF. 1μg of fibrils were analyzed in a 10% SDS-PAGE gel using the C20 antibody (α-Synuclein monomer in higher exposure is shown in cropped gel/blot). No signal was observed for the (mock-P) and the S129A-PFF with the α-Synuclein (phospho Ser) antibody. Human PLK2 (Snk) was not detected in any of the fibrilar material treated with the enzyme. Lysate from SH-SY5Y cells, overexpressing human wt α-Synuclein (wt-ASYN)[3], was used as a positive control. **b** Mouse primary cultures (6div) were treated with P-PFF, S129A-PFF and (mock-P) S129A-PFF for 24 hours. Following Triton-X extraction, internalized fibrils were visualized with the

human specific α-Synuclein antibody 4B12. Treatments with either the mutant S129A- or its phosphorylated equivalent (mock-P) S129A-PFF resulted in a similar pattern of uptake. In contrast, increased internalization capacity was observed for the P-PFF. β-actin was used as a loading control. **c** Immunocytochemistry with the rodent specific α-Synuclein antibody (D37A6) in primary mouse cortical cultures treated with P-PFF, S129A-PFF and (mock-P) S129A-PFF for 5 days. Both mutant S129A-PFF and (mock-P) S129A-PFF appeared similarly effective to seed the endogenous α-Synuclein versus PBS control treated neurons. Still P-PFF treatment resulted in significantly increased endogenous α-Synuclein signal compared to the mutant PFF and PBS control treated neurons. Scatter plot present the mean values of fluorescence intensity/cell of two independent experiments in triplicate wells ±SEM. β Tubulin III (Tuj 1) was used as a neuronal marker. TO-PRO-3 (blue) was used as a cell nuclear marker. Statistical significance was obtained by non parametric Kruskal-Wallis test followed by Dunn's post-hoc test. Scale bar represent 25μm. $p<0,0001$

Supplementary Figure S14

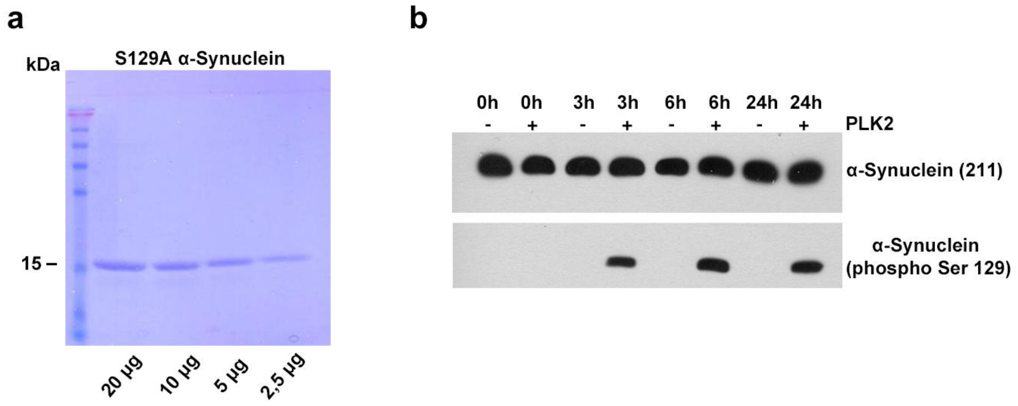

Supplementary Figure S14. a SDS-PAGE was used to characterize the purity of S129A-α-Synuclein. Different amounts of the protein were loaded onto 15% SDS-PAGE and stained with coomassie stain, to examine the purity of the monomeric

protein **b** The phosphorylation of wt α-Synuclein was verified by western blotting using different time points of incubation with PLK2 enzyme. P-α-Synuclein was detectable with the α-Synuclein (phospho Ser129) antibody after 3 hours of incubation. α-Synuclein-211 antibody was used against α-Synuclein monomers

Supplementary video: presents the fluorescence image stacks in 3D reconstruction. Large cytoplasmic inclusions of accumulated P-α-Synuclein (red), surrounding the nucleus of tyrosine-hydroxylase (TH) positive neurons (green) in the SNpc of P-PFF injected animals. TO-PRO-3 (blue) was used as a nuclear marker. Confocal images were captured using a 100x oil immersion objective with 1.5x zoom factor.

Supplementary Materials and Methods

Thioflavin-S (Th-S) fluorescence assay

PFF formation was monitored by Th-S binding. Each sample (10μl) was mixed with 40 μl of Th-S (25 μM) in PBS. Fluorescence was measured in a 384-well black micro-well plate (Nunc, Denmark) using a Victor X3 2030 (Perkin Elmer) microplate reader with excitation and emission wavelengths of 450 nm and 486 nm, respectively.

AspN digestion by filter aided sample preparation

The P-PFF were solubilized by adding 30 μl lysis solution containing 4% SDS, 0.1M DTT and 0.1 M Tris pH 8.5. The sample was then heated to 95 °C for 3 min. The lysed P-PFF were resuspended in 8 M Urea/100 mM Tris-HCl pH 8.5 (UA solution), and the extract were processed according to the Filter Aided Sample Preparation (FASP) protocol[4] using spin filter device with a 10 kDa cut-off (Sartorius, VN01H02). The filter was extensively washed with the urea (UA) solution by serial centrifugations. Thereafter, proteins on the top of the filters were washed three times with 50 mM ammonium bicarbonate and finally digested by adding 0.4 μg of the

protease AspN (mass-spec grade, SIGMA) in 80 μL of a 50 mM ammonium bicarbonate solution and incubated overnight at 37 °C. Next day, peptides were eluted by centrifugation completely dried by speed-vac-assisted solvent removal. The eluted peptides were reconstituted in 0.1% formic acid, 2% acetonitrile in water. Peptide concentration was determined by nanodrop absorbance measurement at 280 nm.

LC-MS/MS analysis

The purified peptides were analyzed by HPLC MS/MS using coupled to an LTQ Orbitrap XL Mass spectrometer (Thermo Fisher Scientific, USA) equipped with a nanospray source. Ten μl of the peptide mixtures were pre-concentrated at a flow-rate of 3μl/min for 10 min using a C18 trap column (Acclaim PepMap) and then loaded onto a 50 cm C18 column (75 μm ID, particle size 2 μm, 100Å, Acclaim PepMap RSLC, Thermo Scientific). The binary pumps of the HPLC (RSLCnano, Thermo Scientific) contained solution A (2% (v/v) acetonitrile in 0.1% (v/v) formic acid) and solution B (80% acetonitrile in 0.1% formic acid). The peptides were separated using a linear gradient of 4- 40% B in 110 min at a flow rate of 300 nl/min. The column was placed in an oven operating at 35°C. Full scan MS spectra were acquired in the orbitrap (m/z 300–1600) in profile mode and data-dependent acquisition with neutral loss activated, with the resolution set to 60,000 at m/z 400 and automatic gain control target at 10^6. The six most intense ions were sequentially isolated and multistage activation was used in order to generate a more sequence-informative fragments detected in the linear ion trap. Dynamic exclusion was set to 60 sec. Ions with single charge states were excluded. Lockmass of m/z 445,120025 was used for internal calibration. The software Xcalibur (Thermo Scientific, USA) was used to control the system and acquire the raw files.

Data analysis and database search

Protein identification was performed using Proteome Discoverer 1.4 software (Thermo Fisher Scientific, USA) equipped with SEQUEST HT search engine. Peak lists were searched against the FASTA entry of α-Synuclein with a final peptide mass deviation of 2 ppm, fragmentation mass deviation of 0.8 Da and AspN digestion specificity allowing up to two missed cleavages. Oxidation of methionine, deamidation of aspargine and glutamine, phosphorylation of threonines, serines and tyrosines were set as variable modifications. The peptides were filtered according to their XCorr score versus charge state (XCorr: +2 ≥ 2 and +3 ≥ 2.5). The phosphorylated peptides were evaluated for their phosphorylation status, assignment and scoring of the phospho site probabilities, by the algorithm phosphoRS[5].

Primary cortical cultures

After dissection, brain tissue was incubated in 2mg/mL papain (Sigma-Aldrich) and 50 μg/mL DNAase I (Sigma-Aldrich) in sterile Hank's Balanced Salt Solution (Life Technologies) at 37^0C for 20 minutes. The cortices were washed three times in medium containing 10% fetal bovine serum (FBS) and then dissociated by mechanical trituration with glass pasteur pipettes. Dissociated cells were transferred in fresh culture medium (Neurobasal medium (Gibco, Invitrogen), containing 2% B27 supplement (Gibco, Invitrogen), 0.5 mM L-glutamine, and 1% penicillin/streptomycin) and were plated onto poly-D-lysine-coated (0,1mg/ml, Sigma-Aldrich) plates (Sarstedt, Germany) or glass coverslips (knittel, Germany) at a density of 100,000-170,000 cells/cm^2.

Behavioral analysis

Mice were habituated to the testing room 30 min before tests. Each mouse was handled for 2-3 minutes daily for one week prior to behavioral testing.

Open field

Locomotor activity was assessed as an index of gross motor function in a transparent, acrylic open field arena (The activity of each mouse was tracked by an overhead camera (Panasonic WV-BP332) and analyzed using specialized video tracking software (Ethovision XT 8.5, Noldus, The Netherlands). Locomotor activity was evaluated, as measured by the total distance travelled (cm) in 10 minutes.

Rotarod

Motor coordination and balance were assessed on an accelerating Rotarod (UGO BASILE). Each mouse was placed on the rotating rod with a diameter of 7 cm and after at least one minute of walking face forward on the rotating rod at the speed of 4 rpm (revolutions per minute), 3x5 min trials with accelerating speed from 4 to 40 rpm were conducted with 45 min resting intervals in between. Latency to fall or passively rotate was measured and the mean latency for all 3 trials was used for analysis.

Challenging beam traversal

The challenging beam was used to examine possible deficits in fine motor coordination as previously described[6]. Each mouse was trained to traverse a plexiglass beam consisting of four sections (25 cm each, 1 m total length) of different width (3.5 cm to 0.5 cm by 1 cm increments). After 2 days of training trials a wire mesh (1 cm x 2 cm rectangles) was placed approximately 1 cm above the beam and animals were video-recorded while traversing the grid-surfaced beam for a total of five trials. Errors

per step (number of limb slips through grid surface per step) were measured .The mean errors per step for the four best trials were analyzed.

Pole test

The pole test is typically used to assess fine motor coordination and balance in basal ganglia-related movement disorders in mice[6,7]. Each mouse was placed head-up on top of a vertical wooden pole (50 cm height, 1 cm in diameter) placed in its home cage as previously described[6]. The animals received two days of training, each consisting of 5 trials. On the test day, time to orient downward (t-turn, sec) and time to descend the pole (t-total, sec) were calculated. Mean time of 5 trials was analyzed for each animal.

Grip strength

A digital grip strength meter (Ahlborn, Almemo-2450) was used to measure grip strength (g) of fore- and hindlimbs. The average of three trials was calculated.

References

1 Majbour, N. K. *et al.* Oligomeric and phosphorylated alpha-synuclein as potential CSF biomarkers for Parkinson's disease. *Molecular neurodegeneration* **11**, 7, doi:10.1186/s13024-016-0072-9 (2016).

2 Bennett, M. L. *et al.* New tools for studying microglia in the mouse and human CNS. *Proceedings of the National Academy of Sciences of the United States of America* **113**, E1738-1746, doi:10.1073/pnas.1525528113 (2016).

3 Vekrellis, K., Xilouri, M., Emmanouilidou, E. & Stefanis, L. Inducible over-expression of wild type alpha-synuclein in human neuronal cells leads to caspase-dependent non-apoptotic death. *Journal of neurochemistry* **109**, 1348-1362, doi:10.1111/j.1471-4159.2009.06054.x (2009).

4 Wisniewski, J. R., Zougman, A., Nagaraj, N. & Mann, M. Universal sample preparation method for proteome analysis. *Nature methods* **6**, 359-362, doi:10.1038/nmeth.1322 (2009).

5 Taus, T. *et al.* Universal and confident phosphorylation site localization using phosphoRS. *Journal of proteome research* **10**, 5354-5362, doi:10.1021/pr200611n (2011).

6 Fleming, S. M. *et al.* Early and progressive sensorimotor anomalies in mice overexpressing wild-type human alpha-synuclein. *The Journal of neuroscience : the official journal of the Society for Neuroscience* **24**, 9434-9440, doi:10.1523/JNEUROSCI.3080-04.2004 (2004).

7 Ogawa, N., Hirose, Y., Ohara, S., Ono, T. & Watanabe, Y. A simple quantitative bradykinesia test in MPTP-treated mice. *Research communications in chemical pathology and pharmacology* **50**, 435-441 (1985).
